# Uncovering the Gut–Liver Axis Biomarkers for Predicting Metabolic Burden in Mice

**DOI:** 10.3390/nu15153406

**Published:** 2023-07-31

**Authors:** Guiyan Yang, Rex Liu, Shahbaz Rezaei, Xin Liu, Yu-Jui Yvonne Wan

**Affiliations:** 1 Department of Medical Pathology, Laboratory Medicine in Sacramento, University of California, Davis, CA 95817, USA; yangguiyan1990@hotmail.com; 2 Department of Computer Science, University of California, Davis, CA 95616, USA; rexliu@ucdavis.edu (R.L.); srezaei@ucdavis.edu (S.R.); xinliu@ucdavis.edu (X.L.)

**Keywords:** diet, aging, bile acid, FXR, gut–liver axis, cognitive dysfunction, chronic inflammation, machine learning

## Abstract

Western diet (WD) intake, aging, and inactivation of farnesoid X receptor (FXR) are risk factors for metabolic and chronic inflammation-related health issues ranging from metabolic dysfunction-associated steatotic liver disease (MASLD) to dementia. The progression of MASLD can be escalated when those risks are combined. Inactivation of FXR, the receptor for bile acid (BA), is cancer prone in both humans and mice. The current study used multi-omics including hepatic transcripts, liver, serum, and urine metabolites, hepatic BAs, as well as gut microbiota from mouse models to classify those risks using machine learning. A linear support vector machine with *K*-fold cross-validation was used for classification and feature selection. We have identified that increased urine sucrose alone achieved 91% accuracy in predicting WD intake. Hepatic lithocholic acid and serum pyruvate had 100% and 95% accuracy, respectively, to classify age. Urine metabolites (decreased creatinine and taurine as well as increased succinate) or increased gut bacteria (*Dorea*, *Dehalobacterium*, and *Oscillospira*) could predict FXR deactivation with greater than 90% accuracy. Human disease relevance is partly revealed using the metabolite–disease interaction network. Transcriptomics data were also compared with the human liver disease datasets. WD-reduced hepatic *Cyp39a1* (cytochrome P450 family 39 subfamily a member 1) and increased *Gramd1b* (GRAM domain containing 1B) were also changed in human liver cancer and metabolic liver disease, respectively. Together, our data contribute to the identification of noninvasive biomarkers within the gut–liver axis to predict metabolic status.

## 1. Introduction

The incidence of metabolic diseases is rising due to obesity. Early diagnosis is needed especially when patients are asymptomatic. Western diet (WD) intake, aging, and farnesoid x receptor (FXR) deactivation are risks for metabolic disease development [1,2,3,4,5,6,7,8]. Additionally, all those factors contribute to systemic inflammation thereby affecting neuroplasticity [9]. FXR is one of the most characterized receptors for bile acids (BAs), which play pivotal roles in regulating lipid and carbohydrate metabolism [10]. Lack of FXR induces hepatic steatosis, metabolic dysfunction-associated steatohepatitis (MASH), and liver tumors spontaneously as mice age [5,7]. Additionally, by deactivating the transcriptional activity of FXR, both aging and WD intake induce the development of metabolic disorders and chronic inflammation [5,10,11]. Furthermore, when these risk factors are combined, the development of MASH and liver carcinogenesis is facilitated [6,7,12]. Similarly, patients with MASH, cirrhosis, or hepatocellular carcinoma (HCC) have reduced expression of FXR [13]. Moreover, aging and dysregulated FXR signaling are also implicated in cognitive function signifying the importance of those factors [14].

Emerging evidence revealed the significance of dysbiosis in contributing to chronic systemic inflammation and the development of metabolic disease via the gut–liver brain axis. Because BAs are generated by the metabolism of hepatic cholesterol using hepatic and bacterial enzymes, dysbiosis is always accompanied by dysregulated BA synthesis [6,7,12]. Additionally, via the diet–gut–brain axis, dysbiosis and dysregulated BA synthesis also have a negative impact on neuroplasticity [15,16,17,18,19]. Furthermore, the importance of the gut–liver–brain axis has been revealed in the development of hepatic encephalopathy [19]. Thus, it is important to discover microbes and metabolites within the gut–liver axis as biomarkers to predict metabolic burden influenced by diet, aging, and FXR functionality.

Computational modeling of multi-omics data can improve the depth and accuracy of omics analysis [20]. Omics data and machine learning approaches have been used for biomarker identification in metabolic liver disease development [21]. In this study, we used mouse datasets including hepatic transcriptomics and metabolomes from multiple sources including the liver, serum, and urine, as well as hepatic BAs, and gut microbiota, to identify predictors for risks of metabolic liver disease development. Our data showed different metabolites from different specimens have a specific power to predict dietary intake or age. Interestingly, the gut microbiota has the best-predicting power to classify FXR functionality. Together, the uncovered molecular signatures within the gut–liver axis might serve as biomarkers for metabolic status.

## 2. Materials and Methods

### 2.1. Mouse Models and Data Collection

Specific pathogen-free male wild-type (WT) and FXR KO mice [1] were fed on either a healthy control diet (CD TD.140415; 5.2% fat, 12% sucrose, and 0.01% cholesterol, *w*/*w*, Harlan Teklad, Madison, WI, USA) or a Western diet (WD, TD.140414; 21.2% fat, 34% sucrose, and 0.2% cholesterol, *w*/*w*, Harlan Teklad, Madison, WI, USA) since weaning and euthanized at the age of 5, 10, and 15 months. Mice were housed in 3–4 per cage in a temperature (24 °C) and light-controlled (12 h light on and off cycle) facility. Multi-omics data were derived from mice with characterized phenotypes [6,7,12,22,23]. The sample size and experimental groups are summarized in Supplementary Table S1. Experiments were performed in accordance with the NIH Guide for the Care and Use of Laboratory Animals under protocols (#21701) approved by the Institutional Animal Care and Use Committee of the University of California, Davis.

### 2.2. Analysis of Liver, Serum, and Urine Metabolites

Hepatic metabolites were measured by gas chromatography–time-of-flight mass spectrometry (GC-TOF-MS) using a sample size of 6 per group by the West Coast Metabolomics Center at the University of California, Davis. Serum and urine metabolites were quantified by NMR, and their concentrations were normalized by log transformation to reduce batch effects [24].

### 2.3. RNA Sequencing and Data Processing

Hepatic bulk RNA was used for library preparation followed by sequencing, which was conducted by Novogene Co., Ltd. (Sacramento, CA, USA). Raw FASTQ data were mapped to GRCm39 (GENCODE release M27) and quantified using Salmon (version 1.4.0) [23]. DESeq2 (version 1.18) with the lfcShrink function in R 4.03 was used to identify differentially expressed genes (DEGs) [25]. Genes with false discovery rate (FDR) corrected *p*-value < 0.05 and absolute fold change ≥2 were considered as DEGs. 

### 2.4. Microbiota Data Analysis

Bacterial DNA from cecal content was extracted for 16S rRNA sequencing [6]. The 16S rRNA gene (V4 region) was amplified and sequenced using Illumina MiSeq. Sequence reads were analyzed by QIIME [6,7]. 

### 2.5. Bile Acid Quantification

Hepatic BAs were quantified using a ProminenceTM UFLC system (Shimadzu, Kyoto, Japan) coupled to an API 4000 QTRAPTM mass spectrometer (AB Sciex) operated in a negative ionization mode [6]. 

### 2.6. Human Datasets

RNA sequencing data of 371 human HCCs and 50 normal livers were obtained from The Cancer Genome Atlas (TCGA) database. Transcriptomic data from steatotic liver disease (SLD) and MASH, with or without fibrosis, cohorts were from the Gene Expression Omnibus (GEO) database (GSE 135251). 

### 2.7. Transcriptomic Feature Selection

Because the number of detected hepatic transcripts was much bigger than the sample size, feature selection was conducted to reduce noise in the dataset and speed up the training process [26]. Features were selected based on differences between groups with statistical significance (*p* < 0.05) and fold change greater or equal to 2. To study dietary effects, 42 transcripts that commonly changed their expression levels in all 3 age groups (5, 10, and 15 months) were selected. Irrespective of diets, 256 transcripts differentially found between 5- and 15-month-old mice were selected. In addition, 105 transcripts differentially expressed in the livers of FXR KO and WT mice, irrespective of dietary and age differences, were included. 

### 2.8. Machine Learning Models

Specific binary classification targets were established, leveraging data obtained from mouse models. These targets comprised diet, age, and FXR functionality.

Subsequently, classification models tailored to each target were constructed using marker panels selected in Section 2.7. To assess their efficacy, these models were subjected to the rigorous evaluation framework of *K*-fold cross-validation. Support vector machine (SVM) is one of the most robust classical machine learning algorithms, which constructs a set of hyperplanes in a high dimensional space to separate classes with the largest margin between the boundary of each class [27,28]. In comparison with other methods, such as principal component analysis (PCA) and canonical correlation analysis (CCA), SVM allows us to extract and evaluate feature importance, enabling a better understanding of the contribution of each feature to the classification decision. Linear SVM is an SVM approach that has garnered wide adoption in healthcare applications for its superior performance in diverse contexts [27,28,29,30,31]. Linear SVM provides better interpretability by revealing the relationship between features and final prediction [32]. Initial analysis indicated that linear SVM exhibited higher prediction accuracy compared with non-linear alternatives and others such as logistic regression, linear regression, random forest, and decision tree. Furthermore, linear SVM showcased particular suitability for analyzing datasets with limited sample sizes [26]. Consequently, linear SVM was deemed the most appropriate choice for constructing risk prediction models for this study. 

To accurately gauge the predictive performance of the linear SVM classification model, *K*-fold cross-validation was employed. Sixteen-fold cross-validation was used for transcriptomic data, and twenty-fold cross-validation was used for other omics data. *K*-fold cross-validation serves as a resampling technique widely employed to assess the efficacy of machine learning models. By employing this technique, biases and variances inherent in the evaluation metric are mitigated, providing a more reliable estimate of model performance. The process involved randomly shuffling the data and splitting it into K groups. Each algorithm was trained on K-1 groups while utilizing the remaining group as the test set. This process was iterated K times, ensuring that each group served as the test set at least once. Ultimately, the mean classification accuracy and standard deviation were computed across all cross-validation runs, offering a comprehensive assessment of the algorithm’s performance on the dataset.

Upon completing the machine learning algorithm selection and validation process, the focus shifted towards unearthing insights into the effect of each marker. This involved a meticulous examination of the coefficients associated with each marker within the linear SVM classifier. By calculating the associated coefficient, which corresponds to the orthogonal vector coordinate of the hyperplane, the effect of each marker on the final prediction of the model was discerned [32,33]. These coefficients were subsequently ranked, providing insights into the relative effect of each marker for the classification task (the higher the more important).

Furthermore, the model’s performance was assessed by systematically testing different combinations of markers, encompassing a range from the highest-ranked marker to including all markers. This comprehensive evaluation approach aimed to elucidate the collective impact of various marker combinations and their relationship with the model’s prediction accuracy. 

Finally, the findings were presented by identifying the least number of top-ranked features necessary to achieve a prediction accuracy of 90% or higher for the classification targets, namely diet, age, or FXR expression status. The culmination of this effort was a comprehensive assessment of various feature combinations, visually presented using line charts. To facilitate the reproducibility of the research, all the Python scripts used in this study were available at https://anonymous.4open.science/r/Molecular_Markers_for_Metabolic_Disease-F7FE accessed on 19 June 2023.

### 2.9. Pathway and Network Analysis

Kyoto Encyclopedia of Genes and Genomes (KEGG) pathway analysis for metabolites and transcripts was performed using MetaboAnalyst 5.0. The metabolite–disease interaction network in MetaboAnalyst 5.0 was used to explore disease-related metabolites based on Human Metabolome Database. 

### 2.10. Association Analysis

Spearman’s correlation was used to assess the relationship between the predictors of each risk factor in this study. A significant correlation was defined when adjusted * *p* < 0.05 and ** *p* < 0.01 using Hochberg. 

### 2.11. Statistics 

The altered metabolites and bacteria between groups were considered by *p* < 0.05 using unpaired *t*-tests. DEGs were generated using DESeq2. Bar graphs were generated by GraphPad Prism Version 8.0 (San Diego, CA, USA). *p* values: * *p* < 0.05; ** *p* < 0.01; *** *p* < 0.001. 

## 3. Results

### 3.1. Predictors of Differential Diet Intakes

**Figure 1:**
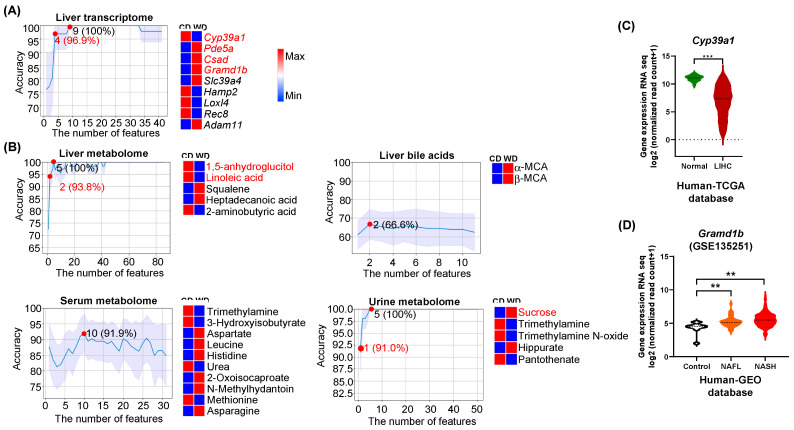
Predictors of differential diet intake based on multi-omics data. Machine learning model generated line charts on the number of features and indicated accuracy using the *K*-fold cross-validation method for (**A**) liver transcriptome and (**B**) metabolomes from the liver, serum, and urine as well as hepatic bile acids. The number of features with predictive accuracy higher than 90% and/or the number of least features that has the highest predicting accuracy is highlighted (red dot). The differences in the relative abundance of predictors between CD and WD groups are shown in heatmaps (blue and red indicate low and high levels, respectively). The order of features in the heatmap is based on the feature’s importance (coefficient value) after feature selection. (**C**) Human HCC patients (*n* = 371) have reduced *Cyp39a1* transcript compared with normal livers (*n* = 50) from the TCGA database. (**D**) A violin plot shows human NAFL/SLD (*n* = 51) and NASH/MASH (*n* = 155) cohorts have higher *Gramd1b* mRNA levels than the controls (*n* = 10) from the GEO database (GSE 135251). Data are expressed as the mean ± SD. ** *p* < 0.01, *** *p* < 0.001.

WD consumption induces MASLD and increases body weight during the experimental time frames (5, 10, and 15 months) [22]. The smallest feature numbers, which could generate the highest prediction accuracy are summarized in Table 1. Nine hepatic transcripts (*Cyp39a1*, *Pde5a*, *Csad*, *Gramd1b*, *Slc39a4*, *Hamp2*, *Loxl4*, *Rec8*, and *Adam11*) classified differential dietary intake with 100% accuracy (Figure 1A). Specifically, downregulated *Cyp39a1* together with upregulated *Pde5a*, *Csad*, and *Gramd1b* had 96.9% accuracy to predict WD intake (Figure 1A). 

The findings were compared with human TCGA (HCC) and GEO (SLD, MASH) databases (GSE 135251). In consistency, the expression of *Cyp39a1*, which is involved in cholesterol clearance through BA synthesis, was downregulated in human HCC compared with normal livers (*p* < 0.001) (Figure 1C). Additionally, *Gramd1b*, a cholesterol transporter, was consistently elevated in SLD and MASH patients compared with healthy controls (*p* < 0.01) (Figure 1D).

In the livers, 5 metabolites (1,5-anhydroglucitol, linoleic acid, 2-aminobutyric acid, squalene, and heptadecanoic acid) had 100% accuracy in predicting differential diet intake (Figure 1B). Decreased 1,5-anhydroglucitol and linoleic acid yielded 93.8% accuracy in classifying diets. Hepatic α-MCA and β-MCA had 66.6% accuracy in distinguishing differential diets (Figure 1B). Moreover, increased aspartate, leucine, histidine, 2-oxoisocaproate, N-methylhydantoin, and asparagine but decreased trimethylamine, 3-hydroxyisobutyrate, urea, and methionine found in the serum yielded 91.9% accuracy to predict WD intake. For urine metabolites, sucrose, trimethylamine, trimethylamine N-oxide, hippurate, and pantothenate achieved 100% accuracy in predicting diet. Increased urine sucrose alone had 91% accuracy in predicting WD intake (Figure 1B). 

Integrated pathway analysis uncovered that serum leucine, methionine, histidine, asparagine, and aspartate were involved in the central carbon metabolism in cancer and aminoacyl-tRNA biosynthesis (Supplementary Figure S1A). Serum aspartate and histidine as well as urine pantothenate were involved in β-alanine metabolism (Supplementary Figure S1A).

Network analysis showed that reduced hepatic 1,5-anhydroglucitol and linoleic acid were related to Alzheimer’s disease (Supplementary Figure S1B). Increased urine sucrose was related to lung cancer. Increased urine TMAO was associated with many diseases including schizophrenia, propionic acidemia, maple syrup urine disease, lung cancer, and dimethylglycine dehydrogenase deficiency (Supplementary Figure S1B).

Spearman’s correlation analysis revealed that urine sucrose was negatively associated with hepatic 1,5-anhydroglucitol and linoleic acid. Interestingly, increased urine sucrose was also negatively associated with the expression levels of *Cyp39a1*, but positively correlated with *Pde5a*, *Gramd1b*, and *Csad*. The decreased serum 3-hydroxyisobutyrate was positively associated with hepatic linoleic acid but negatively correlated with the expression level of *Gramd1b* (Supplementary Figure S2). The key functions or the known roles of those transcripts and metabolites are summarized in Supplementary Table S2 and Table S3, respectively.

### 3.2. Age Classification

**Figure 2:**
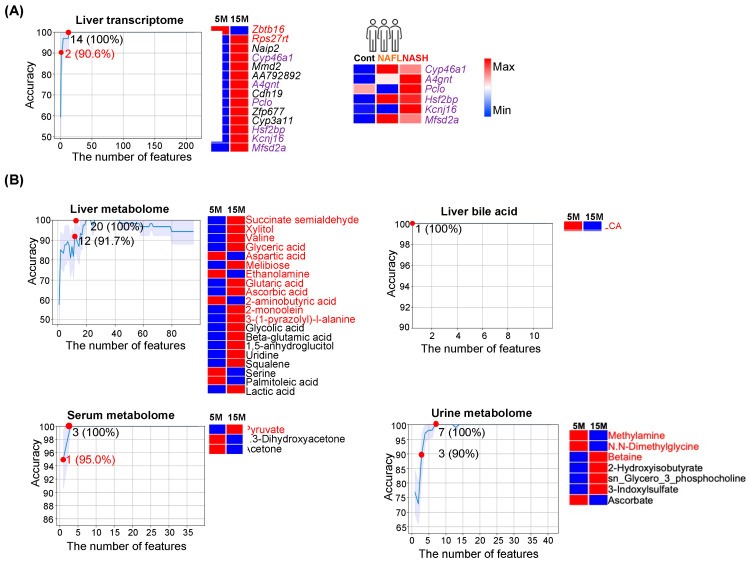
Age classification. The representative line charts show the number of features with the corresponding accuracy in classifying age using (**A**) liver transcriptomes, and (**B**) metabolomes from the liver, serum, urine as well as hepatic bile acids. (**A**) Heatmaps show the level of 14 transcripts with 100% accuracy in the prediction of age and overlapped transcripts in human NAFL/SLD and NASH/MASH cohorts (highlighted in purple). (**B**) Heatmaps show the differences in the relative abundance of metabolites from liver, serum, and urine as well as hepatic bile acids in 5- and 15-month-old mice (blue and red indicate low and high levels, respectively). The number of features with predictive accuracy > 90% or the number of least features that has the highest accuracy are marked (red dots). The order of features in the heatmap is based on the importance of the feature after feature selection.

Under the influence of an unhealthy diet, aging further reduces metabolic efficiency. Thus, there was a temporal effect of WD intake, and 15-month-old WD-fed mice had the most severe MASLD [12]. The machine learning model revealed that downregulated hepatic *Zbtb16* and upregulated *Rps27rt*, *Naip2*, *Cyp46a1*, *Mmd2*, *AA792892*, *A4gnt*, *Cdh19*, *Pclo*, *Zfp677*, *Cyp3a11*, *Hsf2bp*, *Kcnj16*, *Mfsd2a*, yielded 100% accuracy to differentiate 15- vs. 5-month-old mice livers (Figure 2A). Moreover, two transcripts (*Zbtb16* and *Rps27rt*) had 90.6% accuracy to classify the age. 

The disease relevance of those age-related hepatic transcripts was studied using human datasets. In humans, hepatic *CYP46A1*, *A4GNT*, *PCLO*, *HSF2BP*, *KCNJ16*, and *MFSD2A* were also found to be elevated in MASH patients compared with healthy controls (Figure 2A, right panel). 

Among the studied molecular signatures including transcripts and metabolites from different sources, hepatic BAs generated the best-predicting value to differentiate ages as reduced lithocholic acid (LCA) alone achieved 100% accuracy (Figure 2B). In addition, twelve liver metabolites (e.g., glyceric acid, melibiose, glutaric acid, etc.), or one serum metabolite (pyruvate), or three urine metabolites (methylamine, N.N-dimethylglycine, and betaine) each had ≥90% accuracy in classification of chronological age (Figure 2). 

Integrated pathway analysis was performed for age predictors including transcripts and metabolites (Supplementary Figure S3A). The top regulated pathways are ABC transporters (hepatic aspartic acid, valine, xylitol, uridine, and urine betaine), as well as glycine, serine, and threonine metabolism (hepatic glyceric acid and aspartic acid, urine betaine, and serum pyruvate) (Supplementary Figure S3A). 

The disease relevance is elucidated by the metabolite–disease interaction network. In humans, most of the uncovered age-related metabolites were implicated in schizophrenia, Alzheimer’s disease, and lung cancer (Supplementary Figure S3B). 

Correlation analysis showed that hepatic LCA was positively associated with serum concentrations of acetone and 1,3-dihydroxyacetone, but negatively correlated with serum pyruvate (Supplementary Figure S4). Instead of using liver samples, our data revealed that serum metabolites (pyruvate, acetone, and 1,3-dihydroxyacetone) are significant in classifying chronological age.

### 3.3. Predictors for FXR Inactivation

FXR whole-body KO mice develop SLD, MASH, and liver tumors spontaneously with age [5,34]. WD intake facilitates the progression of liver disease development [6,7]. Thus, the inactivation of FXR leads to carcinogenesis within the experimental time frame (i.e., 15 months) [12]. Among the studied groups, 15-month-old WD-fed FXR KO male mice had the most severe hepatic phenotypes, as many of them not only had steatohepatitis but also liver tumors [12]. 

**Figure 3:**
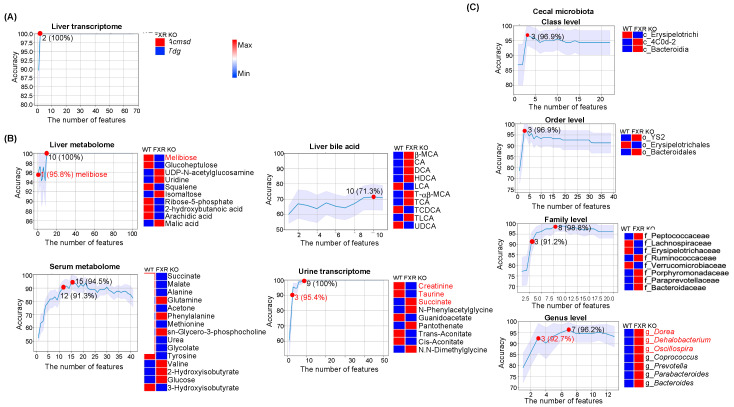
Predictors for FXR expression. Representative line charts show the number of features with indicated accuracy in the prediction of FXR expression status using (**A**) liver transcriptomes, (**B**) metabolomes from the liver, serum, and urine as well as hepatic bile acids, and (**C**) cecal microbiota at different levels. The number of features with predictive accuracy > 90% and/or the number of least features that has the highest accuracy is highlighted (red dot). The differences in the relative abundance of predictors between FXR KO and WT groups are shown in heatmaps (blue and red indicate low and high levels, respectively). The order of features in the heatmap is based on the importance of the feature after feature selection.

For FXR inactivation, 100% accuracy could be achieved based on the expression pattern of two transcripts (upregulated *Acmsd* and downregulated *Tdg*) or ten hepatic metabolites shown in the heatmap (Figure 3A,B). Among ten hepatic metabolites, decreased melibiose had 95.8% accuracy to predict FXR inactivation. However, ten hepatic BAs only gave 71.3% accuracy to predict FXR status. Moreover, twelve serum metabolites (succinate, malate, alanine, glutamine, acetone, phenylalanine, methionine, sn-Glycero-3-phosphocholine, urea, glycolate, tyrosine, valine, 2-hydroxyisobutyrate, glucose, and 3-hydroxyisobutyrate) predicted FXR expression status with 91.3% accuracy (Figure 3B). Further, urine creatinine, taurine, and succinate had 95.4% accuracy in predicting FXR status (Figure 3B). Notably, cecal microbiota at phylum, class, order, family, and genus levels could differentiate FXR KO and WT with > 90% accuracy. Especially, increased *Dorea*, *Dehalobacterium*, and *Oscillospira* at the genus level yielded 92.7% accuracy to differentiate FXR KO vs. WT (Figure 3C). 

Pathway analysis for metabolites shown in Figure 3B revealed that serum glutamine, succinate, malate, phenylalanine, methionine, valine, tyrosine, and alanine were involved in the central carbon metabolism in cancer (Supplementary Figure S5A). The metabolite–disease interaction network showed that urine creatinine, which was reduced due to FXR inactivation was associated with neurological disorders (e.g., Canavan disease and schizophrenia), urinary disorders (e.g., Bartter syndrome, type 2, antenatal and maple syrup urine disease), and metabolic disorders (dimethylglycine dehydrogenase deficiency) (Supplementary Figure S5B). In addition, succinate (succinic acid) was also related to Canavan disease (Supplementary Figure S5B). Urine taurine was associated with maple syrup urine disease (Supplementary Figure S5B). 

Association analysis found that hepatic melibiose was negatively associated with cecal *Dorea*, *Dehalobacterium*, and *Oscillospira* (Supplementary Figure S6B). Additionally, these three bacteria were also negatively associated with hepatic *Tdg* but positively correlated with hepatic *Acmsd*. It indicates that the increased relative abundance of cecal *Dorea*, *Dehalobacterium*, and *Oscillospira* can be a marker of FXR inactivity. The roles of FXR status predictors (transcripts and metabolites) are summarized in Supplementary Tables S2 and S3.

## 4. Discussion

Our data revealed that the performance of multi-omics in each risk prediction model is different based on the predictive accuracy and the number of features (Table 1). Remarkably, urine metabolite (sucrose), serum metabolites (pyruvate, acetone, and 1,3-dihydroxyacetone), and gut bacteria (*Dorea*, *Dehalobacterium*, and *Oscillospira*) can classify (>90% accuracy) dietary patterns, ages, and FXR functional status, respectively. The molecular features that act as metabolic liver disease risk predictors are not only biomarkers for risk factors in mouse models but also related to human diseases. Some features have been reported to be involved in the pathogenesis of human diseases and they maybe also act as treatment targets for human diseases. The information is summarized in Supplementary Tables S2 and S3.

### 4.1. Diet Predictors Relate to Metabolic Liver Disease Development

Among the diet predictors, the downregulation of *Cyp39a1* (24-hydroxycholesterol 7-alpha-hydroxylase) by WD has been proposed as a novel biomarker for poor overall survival of HCC patients [35]. In contrast, *Gramd1b* (GRAM domain containing 1B), a cholesterol transporter, was upregulated in WD-fed mouse livers suggesting cholesterol overload. Consistent with our findings, the expression of hepatic *Gramd1b* is also increased by a high-cholesterol diet, and silencing hepatic Gramd1b in mice suppresses MASH progression [36].

Among the metabolites, reduced hepatic 1,5-anhydroglucitol (an anhydro sugar of D-glucitol) and linoleic acid could predict WD intake with 93.8% accuracy. The 1,5-anhydroglucitol, derived mainly from nearly all foods, is lower in fibrosis stage F3 than in the F0–2 stage in MASLD patients [37]. The concentration of linoleic acid is also decreased in human HCC tissues compared with normal controls [38]. Linoleic acid is the most abundant ω-6 polyunsaturated fatty acid in human diets, human plasma, and membrane lipids [39]. 

To develop noninvasive biomarkers of metabolic liver disease risks, we detected urine metabolites and identified that an increase in urine sucrose could be used to predict WD intake. This is not surprising, as the used WD in our animal experiments contains 37% sucrose. It has been shown that there is a significant correlation between the average urinary sucrose excretion and dietary sucrose intake because of sucrose permeability [40]. 

### 4.2. Features That Classify Ages and Metabolic Liver Diseases

Aging is an inevitable risk factor for most chronic diseases, as it decreases regenerative ability and metabolic processes [41]. Zbtb16 (zinc finger and BTB domain-containing protein 16), a transcription factor and energy metabolism regulator, is downregulated in aged mice. The *Zbtb16-*encoded protein is important in adipogenesis and the control of hepatic gluconeogenesis [42]. In humans, decreased Zbtb16 variants are associated with elevated total and low-density lipoprotein cholesterol in a sex-dependent manner [43].

Age also affects the profile of BAs, which have pivotal roles in metabolism, immunity, and anti-tumorigenesis. Notably, decreased hepatic LCA could predict older age with 100% accuracy. Consistently, LCA has been identified as an anti-aging compound that extends the lifespan of yeast [44]. LCA acts as an agonist of the G-protein-coupled BA receptor named Takeda G protein-coupled receptor 5 (TGR5) in increasing free fatty acid availability through lipolysis and induces mitochondrial fission [45]. As the expression of FXR and TGR5 declines with age, dual agonists for FXR and TGR5 have been shown to delay age-related kidney deterioration in mouse models [46]. In humans, isoforms of LCA (iso-, 3-oxo-, allo-, 3-oxoallo-, and isoallolithocholic acid)-producing bacteria were enriched in centenarians [47]. In rats, dietary conjugated LCA, a mixture of positional and geometric isomers of linoleic acid, alleviates MASLD [48]. Taken together, LCA may be a target for aging-related MASLD treatment. 

Our data revealed that serum pyruvate as well as acetone (a ketone body) and 1,3-dihydroxyacetone (DHA) correlated with hepatic LCA. The potential of reduced serum pyruvate together with increased serum acetone and DHA being a metabolically active young liver, warrants further validation in humans. Serum pyruvate is derived from alanine and α-ketoglutarate converted by the alanine aminotransferase (ALT) and elevated ALT is a diagnostic marker for liver injury. The concentration of serum pyruvate was also elevated by high-fat diet intake [49]. 1,3-Dihydroxyacetone is a 3-carbon reducing sugar produced from glycerol. Acetone is the simplest ketone body and is synthesized from fatty acid oxidation in the livers. Thus, reduced serum acetone likely indicates reduced fatty acid oxidation. Moreover, elevated breath acetone is a biomarker of type 2 diabetes mellitus in the breath analysis [50]. Whether reduced serum acetone can be a biomarker for reduced fatty acid oxidation associated with aging liver also warrants further investigation. 

### 4.3. FXR Inactivation Predictors and Metabolic Liver Diseases

Hepatic transcripts *Acmsd* (aminocarboxymuconate semialdehyde decarboxylase) and *Tdg* (G/T mismatch-specific thymine DNA glycosylase) could differentiate FXR KO from WT. Upregulated *Acmsd* and downregulated *Tdg* in the livers were signatures of FXR inactivation. ACMSD controls cellular NAD^+^ levels in the liver [51]. Inhibition of Acmsd attenuates hepatic steatosis and reduces liver injury in diet-induced MASLD mouse models [52]. TDG (thymine DNA glycosylase) is an enzyme that plays a key role in active DNA demethylation. It is essential for maintaining glucose and BA homeostasis, as depletion of *Tdg* causes dysregulation of FXR signaling and leads to HCC development in mice [53]. 

It is interesting to note that the increased abundance of *Dorea*, *Dehalobacterium*, and *Oscillospira* in cecal content has greater than 90% accuracy in FXR KO prediction. In humans, the abundance of *Dorea* is also increased in MASLD patients compared with healthy controls [54]. *Dehalobacterium* is known to have a negative association with the body mass index [55]. *Oscillospira* is increased in high-fat diet-fed mice compared with normal controls [56].

Urine metabolites also predicted FXR functional status. As a signature of FXR KO, urine creatinine and taurine were decreased while succinate was increased. Urine creatinine reflects muscle mass, and low urine creatinine is associated with cardiovascular disease risk [57]. Taurine is beneficial in alleviating fatty liver disease by promoting energy expenditure and preventing oxidative damage and inflammation [58]. Succinate is an inflammation-induced immunoregulatory metabolite in the macrophages [59], and it is also elevated in inflammation [60]. Thus, the metabolic features that predict FXR inactivation are involved in metabolism and immune responses. 

The main strength of the study is using comprehensive multi-omics data generated within the gut–liver axis to predict diet, age, and FXR functionality. Such approaches would be challenging to perform in humans due to variations. However, the uncovered predictors need to be validated in humans to demonstrate disease relevance. In addition, the data were generated using a specific number of a certain strain of mice, which is standard for basic research. Whether the findings apply to all animal species requires validation.

## 5. Conclusions

Collectively, the study has identified features from different sources that have different predicting power to differentiate risks for metabolic disease development. Urine or gut microbiota biomarkers can be valuable for noninvasive diagnosis of metabolic function status. As WD intake, aging, and FXR inactivation are also implicated in other diseases including dermatitis and dementia [8,9,10], the uncovered risk predictors have multiple disease implications and can be potential biomarkers for early diagnosis of diseases related to diet, age, and FXR expression status. In addition, the uncovered beneficial metabolites linked with intact metabolic status might be used as food supplements. 

## Figures and Tables

**Table 1:** Predictive capabilities of multi-omics in Western diet intake, aging, and FXR inactivation ^1^.

Risk Prediction	Western Diet Intake		Aging		Bile Acid Receptor Inactivation	
	Features	Accuracy	Features	Accuracy	Features	Accuracy
**Hepatic Transcripts**	9 (4)	100% (96.9%)	14 (2)	100% (90.6%)	2	100%
**Metabolites**						
Bile acids (liver)	2	66.6%	1	100%	10	71.3%
Liver	5 (2)	100% (93.8%)	20 (12)	100% (91.7%)	10 (1)	100% (95.8%)
Serum	10	91.9%	3 (1)	100% (95.0%)	15 (12)	94.5% (91.3%)
Urine	5 (1)	100% (91.0%)	7 (3)	100% (90.0%)	9 (3)	100% (95.4%)
**Microbiota**						
Phylum level	8	61.9%	4	70.0%	6	90.2%
Class level	9	62.6%	9	82.8%	3	96.9%
Order level	26	62.5%	13	82.8%	3	96.9%
Family level	10	76.8%	7	80.4%	8 (3)	98.8% (91.2%)
Genus level	6	68.8%	7	82.0%	7 (3)	96.2% (92.7%)

^1^ Multi-omics data analyses were conducted in mice of different ages, diets, and genotypes. The least number of features that has the best predictive performance is shown for each risk factor prediction.

## Data Availability

Hepatic bulk RNA sequencing data are available on the GEO database (https://www.ncbi.nlm.nih.gov/geo/; GSE216375) accessed on 22 February 2023. Bioinformatics and statistical results for hepatic transcriptome used for feature selection were available in a previous study [16]. Phenotypic data have been reported in our previous studies [1,2,6,15,16]. Additional information related to this paper can be requested from the authors. All python scripts used in this study are available at the Github repository (https://anonymous.4open.science/r/Molecular_Markers_for_Metabolic_Disease-F7FE accessed on 15 June 2023).

## Supplementary Materials

The following supporting information can be downloaded at: https://www.mdpi.com/article/10.3390/nu15153406/s1, Figure S1: Functional analysis of diet predictors. (A) Integrated pathway analysis showing pathways for WD-predictors (transcripts and metabolites). The corresponding features for the important pathways are indicated. (B) The network shows that metabolomic predictors of WD intake are associated with human diseases; Figure S2: Spearman’s correlation for WD-predictors from the liver, serum, and urine. Spearman’s correlation, * *p* < 0.05, ** *p* < 0.01; Figure S3: Functional analysis of age-predictors. (A) Integrated pathway analysis for age-predictors (metabolites). (B) Features that can classify ages in association with human diseases; Figure S4: Interaction between features that can be used for chronological age prediction. Spearman’s correlation, * *p* < 0.05, ** *p* < 0.01; Figure S5: Functional analysis of FXR expression predictors. (A) The pathways for metabolites serve as FXR expression predictors. (B) The network shows the interaction between metabolites and diseases for FXR expression predictors; Figure S6: Interactions of FXR expression predictors. Spearman’s correlation between cecal microbiota at the genus level, hepatic transcripts, and metabolites from the liver, serum, and urine. * *p* < 0.05, ** *p* < 0.01; Table S1: The sample information of multi-omics data used for training and validation; Table S2: Summary table of transcriptomic predictors of diet, age, and FXR functionality; Table S3: Summary table of metabolomic predictors of diet, age, and FXR functionality [35,43,48,51,53,61,62,63,64,65,66,67,68,69,70,71,72,73,74,75,76,77,78,79,80,81,82,83,84,85,86,87,88,89,90,91,92,93,94,95,96,97,98,99,100,101,102,103,104,105,106,107,108,109,110,111,112,113,114].

## Abbreviations

Acmsd, aminocarboxymuconate semialdehyde decarboxylase; Adam11, ADAM metallopeptidase domain 11; A4gnt, alpha-1,4-N-acetylglucosaminyltransferase; CD, control diet; Cdh19, cadherin 19; Cyp39a1, cytochrome P450 family 39 subfamily a member 1; Cyp3a11, cytochrome P450 family 3 subfamily a member; Cyp46a1, cytochrome P450 family 46 subfamily a member 1; Csad, cysteine sulfinic acid decarboxylase; DEGs, differentially expressed genes; FXR, farnesoid X receptor; GEO, Gene Expression Omnibus; Gramd1b, GRAM domain containing 1B; Hamp2, hepcidin antimicrobial peptide 2; HCC, hepatocellular carcinoma; Hsf2bp, heat shock transcription factor 2 binding protein; Kcnj16, potassium inwardly rectifying channel subfamily J member 16; Loxl4, lysyl oxidase like 4; ML, machine learning; Mfsd2a, major facilitator superfamily domain containing 2A; Mmd2, monocyte to macrophage differentiation associated 2; MASLD (previous nomenclature is NAFLD (nonalcoholic fatty liver disease)), metabolic dysfunction-associated steatotic liver disease; MASH (previous nomenclature is NASH (nonalcoholic steatohepatitis)), metabolic dysfunction-associated steatohepatitis; Naip2, NLR family apoptosis inhibitory protein; Pclo, piccolo presynaptic cytomatrix protein; Pde5a, phosphodiesterase 5A; Rec8, REC8 meiotic recombination protein; Rps27rt, Ribosomal Protein S27; SVM, support vector machine; Slc39a4, solute carrier family 39 member 4; SLD (previous nomenclature is NAFL [nonalcoholic fatty liver]), steatotic liver disease; Tdg, thymine DNA gycosylase; TGR5, Takeda G protein-coupled receptor 5; WD, Western diet; Zbtb16, zinc finger and BTB domain containing 16; Zfp677, zinc finger protein.
