# Supplementary material for: Uncovering the Gut–Liver Axis Biomarkers for Predicting Metabolic Burden in Mice
Source: Nutrients. 2023 Jul 31;15(15):3406. doi: 10.3390/nu15153406 (PMC10421148; doi:10.3390/nu15153406)
Supplement: Supplementary file 1 [file nutrients-15-03406-s001.zip › nutrients-2490498-supplementary.pdf]

Figure S1

(A)

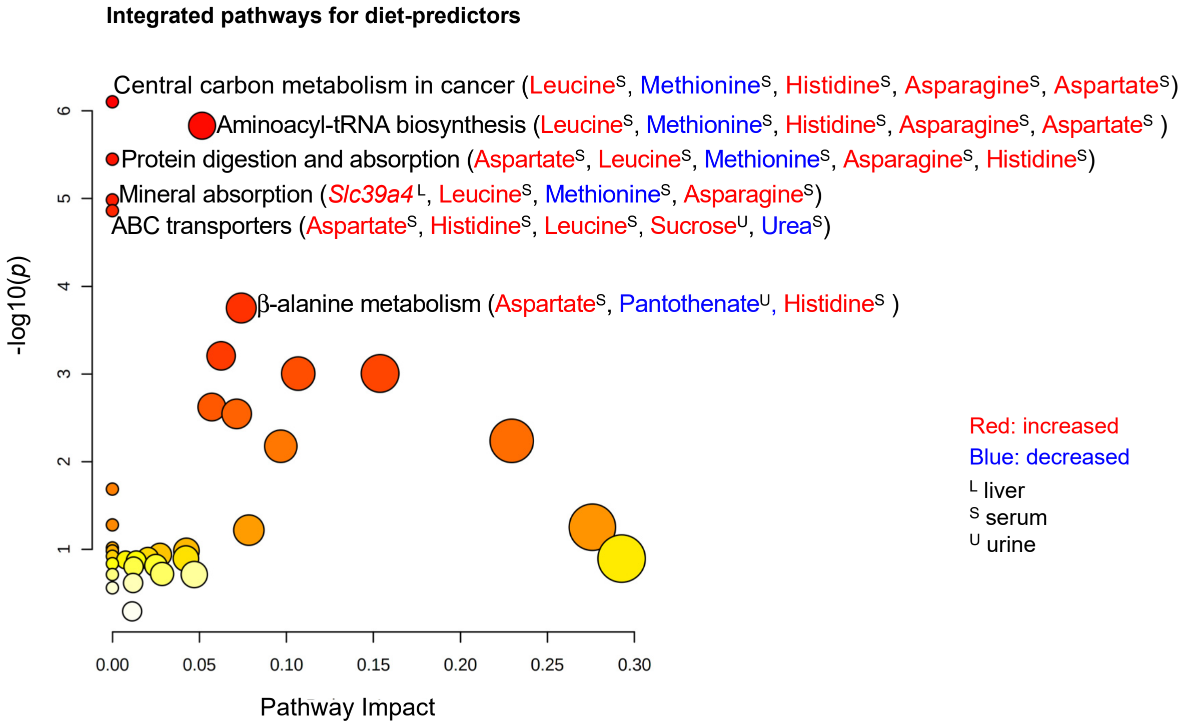

Integrated pathways for diet-predictors
Central carbon metabolism in cancer (Leucine^S, Methionine^S, Histidine^S, Asparagine^S, Aspartate^S)
Aminoacyl-tRNA biosynthesis (Leucine^S, Methionine^S, Histidine^S, Asparagine^S, Aspartate^S)
Protein digestion and absorption (Aspartate^S, Leucine^S, Methionine^S, Asparagine^S, Histidine^S)
Mineral absorption (Slc39a4^L, Leucine^S, Methionine^S, Asparagine^S)
ABC transporters (Aspartate^S, Histidine^S, Leucine^S, Sucrose^U, Urea^S)
β-alanine metabolism (Aspartate^S, Pantothenate^U, Histidine^S)
-log10(p)
Pathway Impact
0.00
0.05
0.10
0.15
0.20
0.25
0.30
1
2
3
4
5
6
Red: increased
Blue: decreased
^L liver
^S serum
^U urine

(B)

Diet predictors
Human diseases-related metabolites

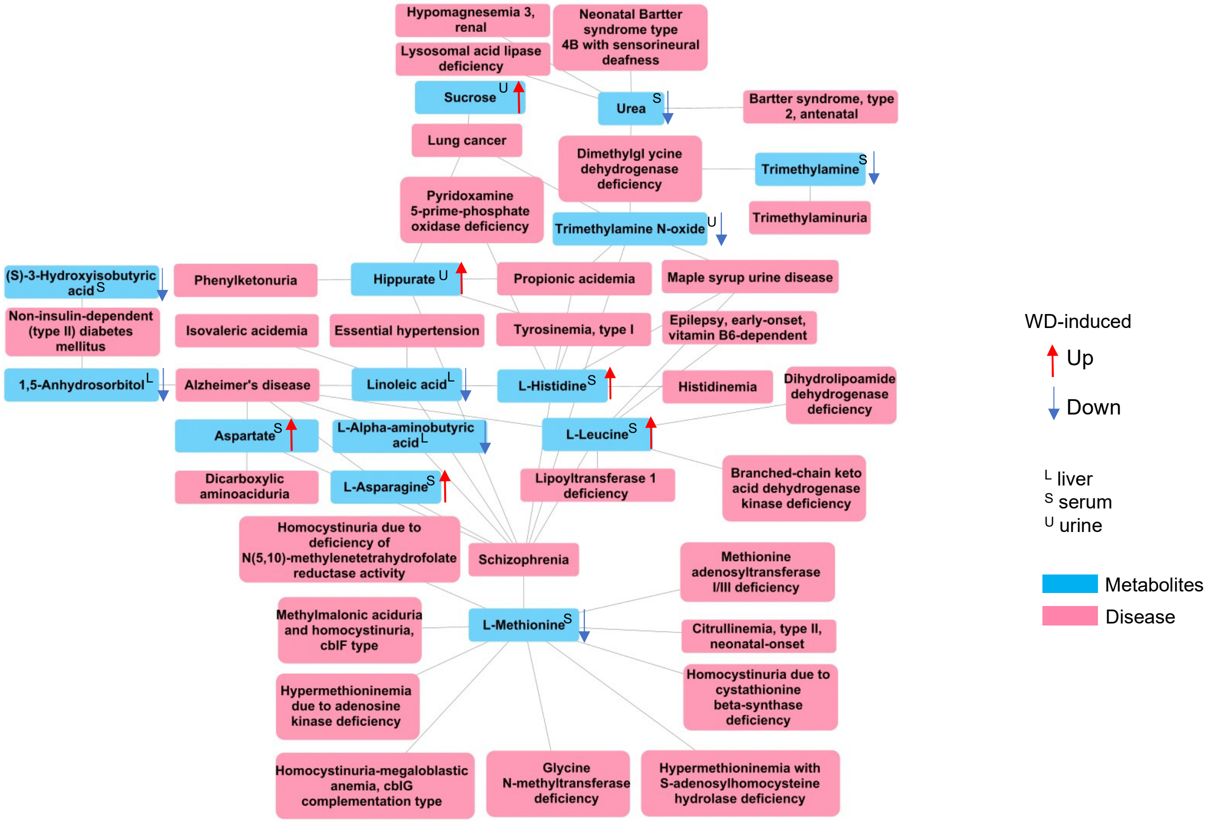

Figure S1. Functional analysis of diet predictors. (A) Integrated pathway analysis showing pathways for WD-predictors (transcripts and metabolites). The corresponding features for the important pathways are indicated. (B) The network shows that metabolomic predictors of WD intake are associated with human diseases.

Figure S2

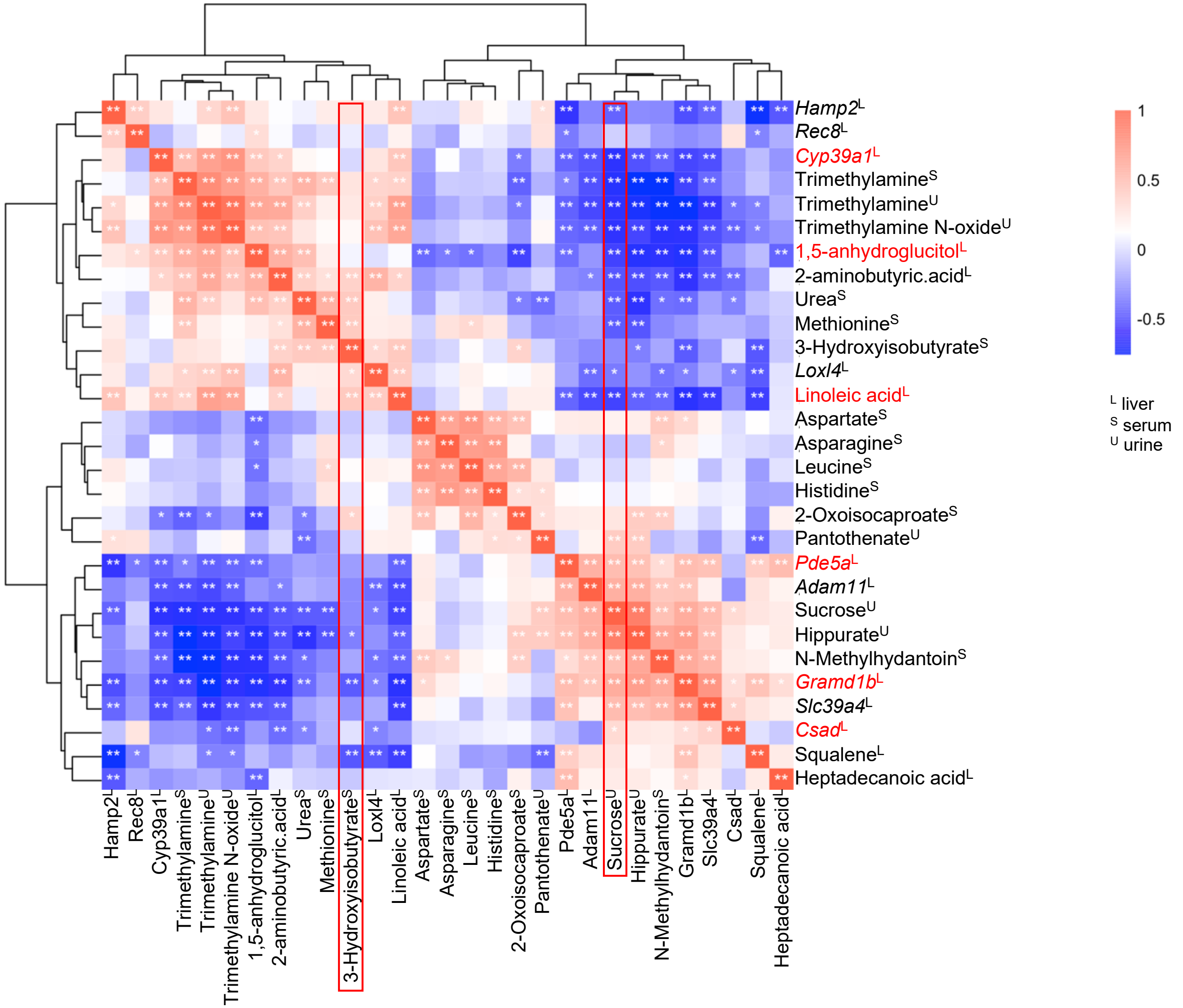

Hamp2L
Rec8L
Cyp39a1L
TrimethylamineS
TrimethylamineU
Trimethylamine N-oxideU
1,5-anhydroglucitolL
2-aminobutyric.acidL
UreaS
MethionineS
3-HydroxyisobutyrateS
Loxl4L
Linoleic acidL
AspartateS
AsparagineS
LeucineS
HistidineS
2-OxoisocaproateS
PantothenateU
Pde5aL
Adam11L
SucroseU
HippurateU
N-MethylhydantoinS
Gramd1bL
Slc39a4L
CsadL
SqualeneL
Heptadecanoic acidL
1
0.5
0
-0.5
L liver
S serum
U urine

Figure S2. Spearman's correlation for WD-predictors from the liver, serum, and urine. Spearman's correlation, **p* < 0.05, ***p* < 0.01.

Figure S3

(A)

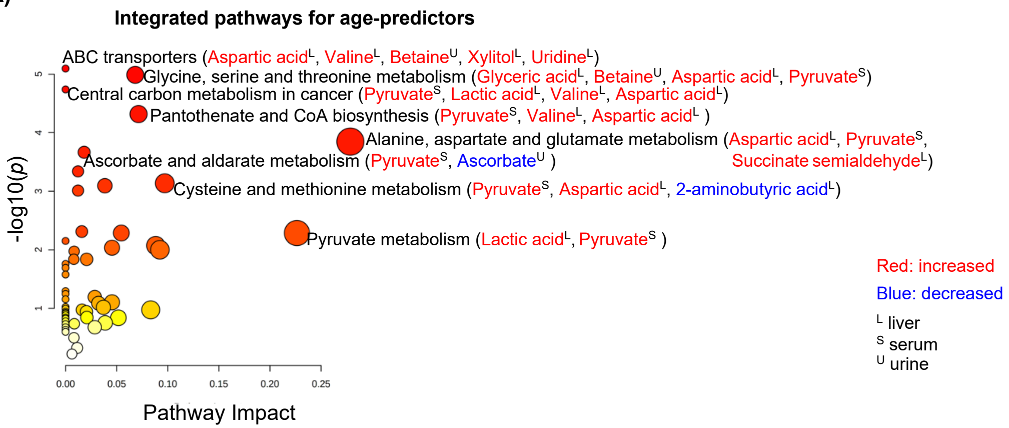

(B)

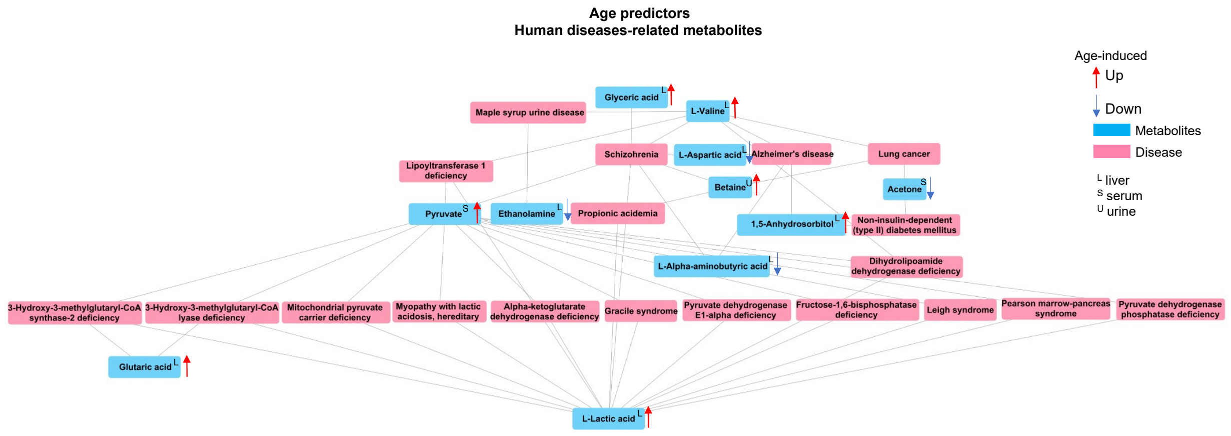

Figure S3. Functional analysis of age-predictors. (A) Integrated pathway analysis for age-predictors (metabolites). (B) Features that can classify ages in association with human diseases.

Figure S4

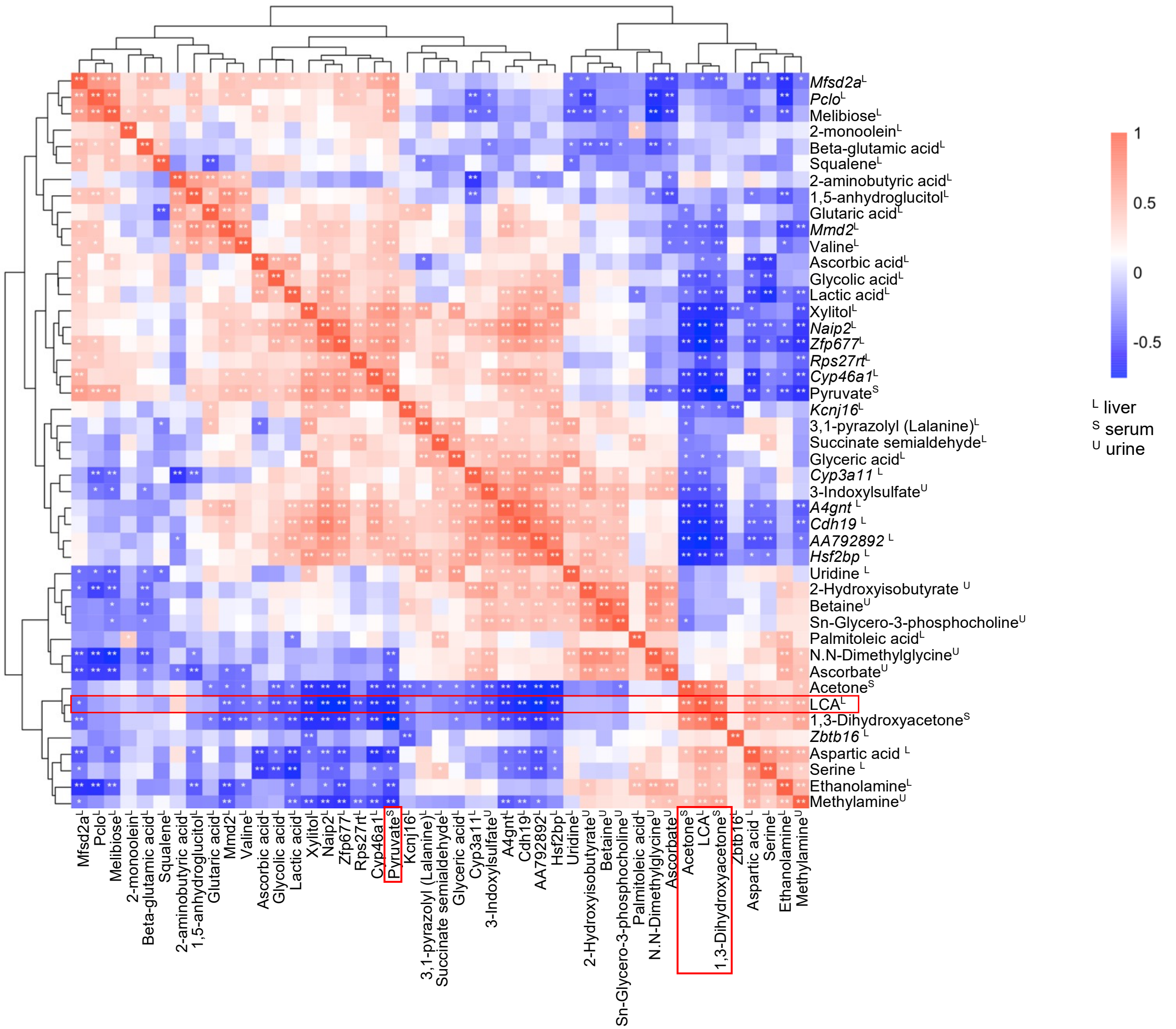

Mfsd2a L
Pclo L
Melibiose L
2-monoolein L
Beta-glutamic acid L
Squalene L
2-aminobutyric acid L
1,5-anhydroglucitol L
Glutaric acid L
Mmd2 L
Valine L
Ascorbic acid L
Glycolic acid L
Lactic acid L
Xylitol L
Naip2 L
Zfp677 L
Rps27rt L
Cyp46a1 L
Pyruvate S
Kcnj16 L
3,1-pyrazolyl (Lalanine) L
Succinate semialdehyde L
Glyceric acid L
Cyp3a11 L
3-Indoxylsulfate U
A4gnt L
Cdh19 L
AA792892 L
Hsf2bp L
Uridine L
2-Hydroxyisobutyrate U
Betaine U
Sn-Glycero-3-phosphocholine U
Palmitoleic acid L
N.N-Dimethylglycine U
Ascorbate U
Acetone S
LCA L
1,3-Dihydroxyacetone S
Zbtb16 L
Aspartic acid L
Serine L
Ethanolamine L
Methylamine U
1
0.5
0
-0.5
L liver
S serum
U urine

Figure S4. Interaction between features that can be used for chronological age prediction. Spearman's correlation, *$p < 0.05$, **$p < 0.01$.

Figure S5

(A)

Integrated pathways for FXR inactivation-predictors

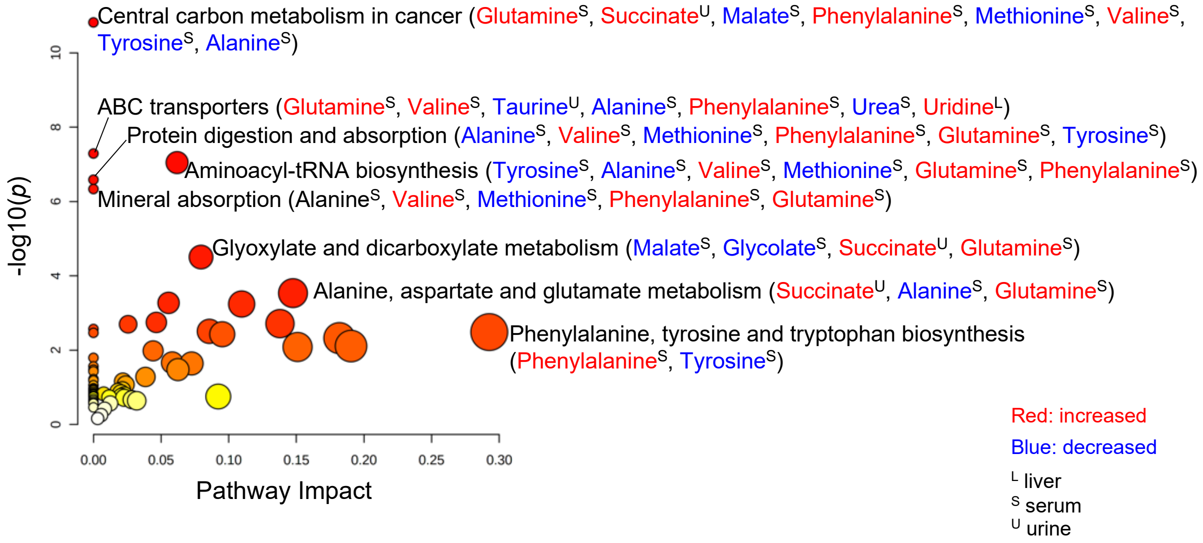

(B)

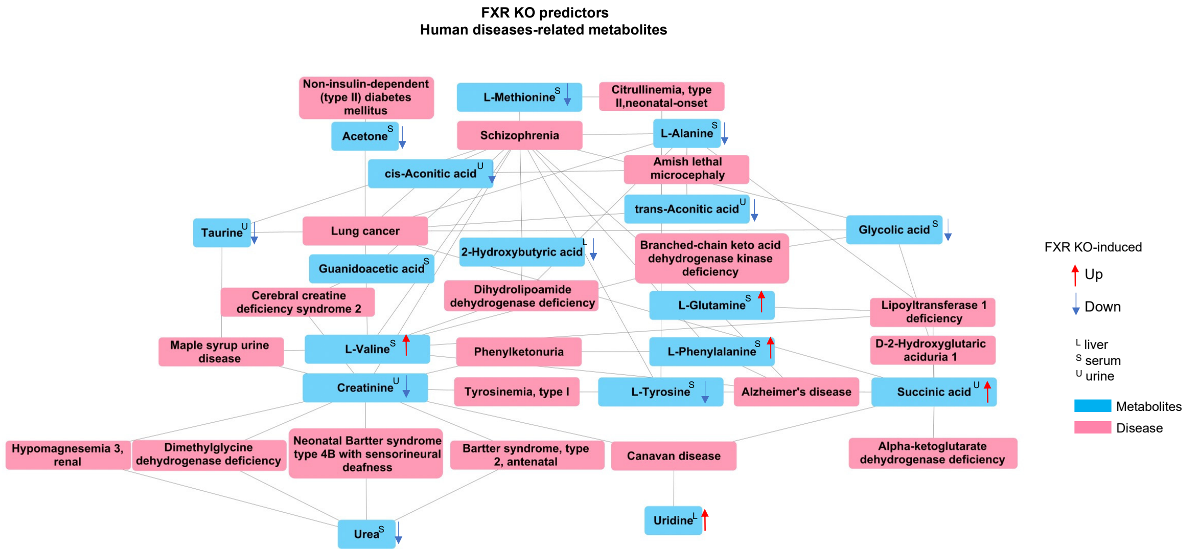

Figure S5. Functional analysis of FXR expression predictors. (A) The pathways for metabolites serve as FXR expression predictors. (B) The network shows the interaction between metabolites and diseases for FXR expression predictors.

Figure S6

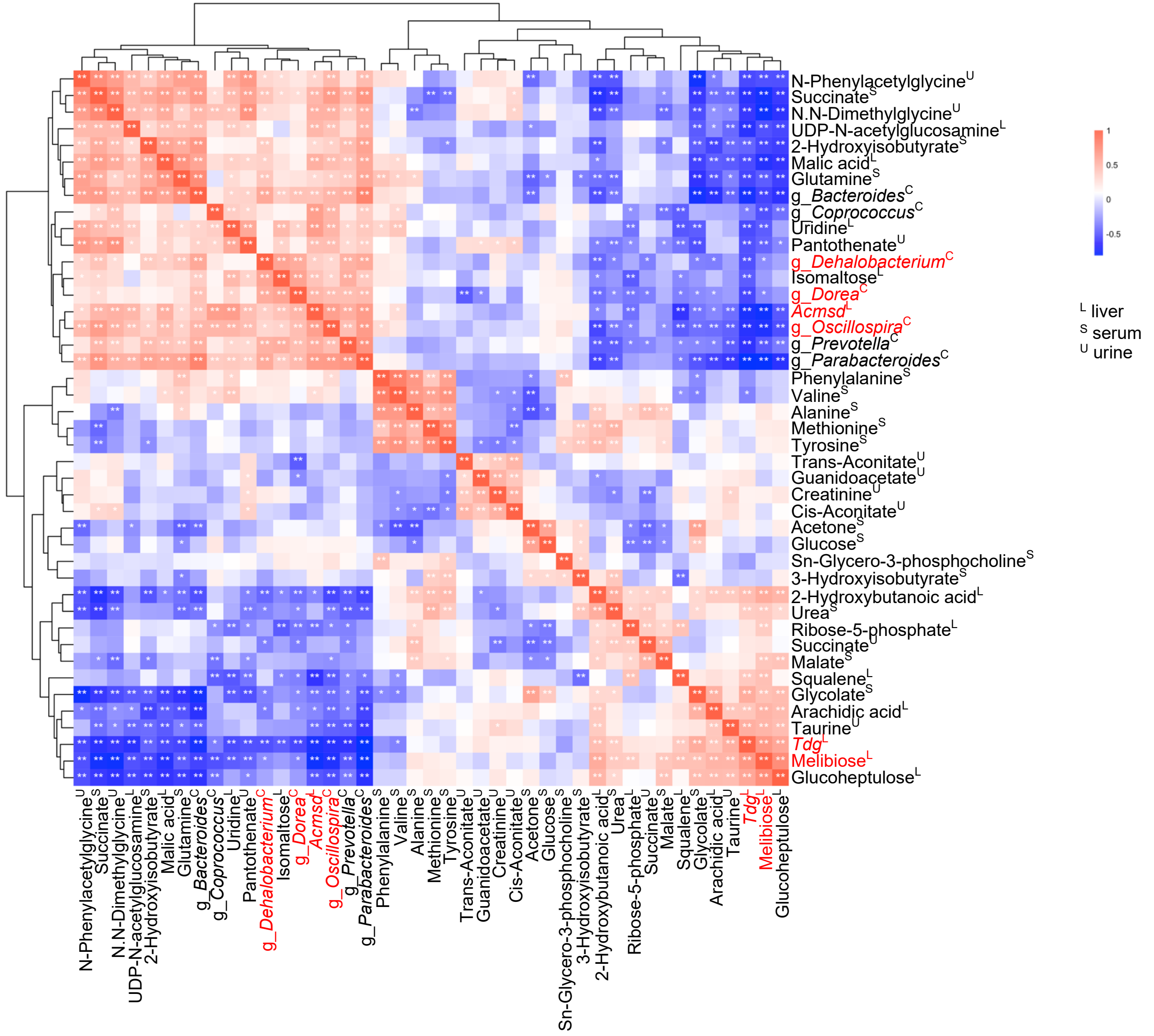

N-Phenylacetylglycine U
Succinate S
N.N-Dimethylglycine U
UDP-N-acetylglucosamine L
2-Hydroxyisobutyrate S
Malic acid L
Glutamine S
g_Bacteroides C
g_Coprococcus C
Uridine L
Pantothenate U
g_Dehalobacterium C
Isomaltose L
g_Dorea C
Acmsd L
g_Oscillospira C
g_Prevotella C
g_Parabacteroides C
Phenylalanine S
Valine S
Alanine S
Methionine S
Tyrosine S
Trans-Aconitate U
Guanidoacetate U
Creatinine U
Cis-Aconitate U
Acetone S
Glucose S
Sn-Glycero-3-phosphocholine S
3-Hydroxyisobutyrate S
2-Hydroxybutanoic acid L
Urea S
Ribose-5-phosphate L
Succinate U
Malate S
Squalene L
Glycolate S
Arachidic acid L
Taurine U
Tdg L
Melibiose L
Glucoheptulose L
1
0.5
0
-0.5
L liver
S serum
U urine

Figure S6. Interactions of FXR expression predictors. Spearman's correlation between cecal microbiota at the genus level, hepatic transcripts, and metabolites from the liver, serum, and urine. *$p < 0.05$, **$p < 0.01$.

Table S1. Samples for multi-omics data from the mouse model.

Omics	Number of mice	Age (month)	Diet	Genotype
**Hepatic transcripts**	48	5, 10, 15	CD, WD	WT, FXR KO
**Metabolites**				
Bile acids (liver)	186	5, 10, 15	CD, WD	WT, FXR KO
Liver	72	5, 10, 15	CD, WD	WT, FXR KO
Serum	122	5, 10, 15	CD, WD	WT, FXR KO
Urine	157	5, 10, 15	CD, WD	WT, FXR KO
**Microbiota**	163	5, 10, 15	CD, WD	WT, FXR KO

Table S2. Hepatic transcripts that classify diet, age, and FXR activity.

Transcript name	Protein name	Functions	Changes	Diseases implications
Cyp39a1	24-hydroxycholesterol 7-alpha-hydroxylase	Steroid metabolism; cholesterol degradation; lipid metabolism; bile acid biosynthesis	Western diet (Down)	CYP39A1 is an HCC suppressor in humans [1].<br>CYP39A1 is an HCC biomarker [2].
Pde5a	cGMP-specific 3',5'-cyclic phosphodiesterase	Purine metabolism, plays a role in signal transduction by regulating the intracellular concentration of cyclic nucleotides	Western diet (Up)	-
Csad	Cysteine sulfinic acid decarboxylase	Organosulfur biosynthesis; taurine biosynthesis	Western diet (Up)	CSAD is protective in NAFLD [3].
Gramd1b	Protein Aster-B	Cholesterol transporter that mediates non-vesicular transport of cholesterol from the plasma membrane to the endoplasmic reticulum	Western diet (Up)	-
Slc39a4	Zinc transporter ZIP4	ZIP4 (SLC39A4) may play a role in the acquisition of zinc by hepatocellular carcinomas, leading to repressed apoptosis, enhanced growth rate and enhanced invasive behavior [4].	Western diet (Up)	SLC39A4 is involved in the pathogenesis of acrodermatitis enteropathica, zinc-deficiency type [5–7].<br>SLC39A4 expression is activated in HCC [4].
Hamp2	Hepcidin-2	Antimicrobial	Western diet (Down)	-
Loxl4	Lysyl oxidase homolog 4	Crosslinking of collagen fibrils; Elastic fibre formation	Western diet (Down)	LOXL4 expression is increased during the liver carcinogenesis [8].

Rec8	Meiotic recombination protein REC8 homolog	Meiotic synapsis	Western diet (Down)	REC8 promotes tumor migration, invasion, and angiogenesis in human HCC [9].
Adam11	Disintegrin and metalloproteinase domain-containing protein 11	LGI (leucine-rich glioma inactivated)-ADAM interactions	Western diet (Up)	-
Zbtb16	Zinc finger and BTB domain-containing protein 16	Energy metabolism regulator	Aging (Down)	ZBTB16 associate with increased obesity-related parameters and elevated total and low-density lipoprotein cholesterol [10].
Rps27rt	Ribosomal Protein S27	RNA binding and structural constituent of ribosome	Aging (Up)	-
Naip2	Baculoviral IAP repeat-containing protein 1b	Inflammatory response; Innate immunity; Apoptosis	Aging (Up)	-
Cyp46a1	Cholesterol 24-hydroxylase	Steroid metabolism; cholesterol degradation; Lipid metabolism; C21-steroid hormone metabolism	Aging (Up)	*Cyp46* gene levels were higher in the hippocampus of elderly humans and in patients with certain pathological, neurodegenerative conditions [11].
Mmd2	Monocyte to macrophage differentiation factor 2	Positive regulation of Ras protein signal transduction	Aging (Up)	-
AA792892	Expressed sequence AA792892		Aging (Up)	-
A4gnt	Alpha-1,4-N-acetylglucosaminyltransferase	O-linked glycosylation of mucins	Aging (Up)	-

Cdh19	Cadherin-19	Wnt signaling pathway; Cadherin signaling pathway	Aging (Up)	-
Pclo	Protein piccolo	Component of the presynaptic cytoskeletal matrix, involved in regulation of presynaptic proteins and synaptic vesicles	Aging (Up)	Loss of PCLO causes pontocerebellar hypoplasia 3 in humans [12];<br>*Pclo* is highly mutated as cancer driver gene in hepatitis B virus-related HCC patients [13].
Zfp677	Uncharacterized protein	-	Aging (Up)	-
Cyp3a11	Cytochrome P450 3A11	Xenobiotics; Aflatoxin activation and detoxification; Biosynthesis of maresin-like SPMs	Aging (Up)	The mRNA expression of hepatic *Cyp3a11* is increased high-fat and high-sucrose induced NAFLD mouse model [14].
Hsf2bp	Heat shock factor 2-binding protein	Double-strand break repair involved in meiotic recombination	Aging (Up)	-
Kcnj16	Inward rectifier potassium channel 16	Potassium transport channels; Activation of G protein gated Potassium channels; Inhibition of voltage gated Ca2+ channels via Gbeta/gamma subunits	Aging (Up)	*Kcnj16* level is lower in HCC tumor tissues compared to adjacent tissues from HCC patients [15]. *Kcnj16* mutation cause a novel tubulopathy with hypokalemia, salt wasting, disturbed acid-base homeostasis, and sensorineural deafness [16].
Mfsd2a	Sodium-dependent lysophosphatidylcholine symporter 1	Glycerophospholipid biosynthesis	Aging (Up)	The MFSD2A level in HCC patients is lower than in healthy controls [17].

Acmsd	2-amino-3-carboxymuconate-6-semialdehyde decarboxylase	Secondary metabolite metabolism; quinolate metabolism	FXR KO (Up)	Inhibiting ACMSD protects liver injury in mouse models [18].
Tdg	G/T mismatch-specific thymine DNA glycosylase	DNA demethylation	FXR KO (Down)	Conditional knockout of Tdg causes HCC in a mouse model [19].

HCC, hepatocellular carcinoma; NAFLD, nonalcoholic liver disease; -, unknown.

References

1. Ji F, Zhang J, Liu N, et al. Blocking hepatocarcinogenesis by a cytochrome P450 family member with female-preferential expression. Gut 2022;71:2313-2324.
2. Li D, Yu T, Hu J, et al. Downregulation of CYP39A1 serves as a novel biomarker in hepatocellular carcinoma with worse clinical outcome. Oxid Med Cell Longev 2021;2021:5175581.
3. Tan R, Li J, Liu L, et al. CSAD ameliorates lipid accumulation in high-fat diet-fed mice. Int J Mol Sci 2022;23.
4. Weaver BP, Zhang Y, Hiscox S, et al. Zip4 (Slc39a4) expression is activated in hepatocellular carcinomas and functions to repress apoptosis, enhance cell cycle and increase migration. PLoS One 2010;5.
5. Wang K, Zhou B, Kuo YM, et al. A novel member of a zinc transporter family is defective in acrodermatitis enteropathica. Am J Hum Genet 2002;71:66-73.
6. Küry S, Dréno B, Bézieau S, et al. Identification of SLC39A4, a gene involved in acrodermatitis enteropathica. Nat Genet 2002;31:239-240.
7. Nakano A, Nakano H, Nomura K, et al. Novel SLC39A4 mutations in acrodermatitis enteropathica. J Invest Dermatol 2003;120:963-966.
8. Tan HY, Wang N, Zhang C, et al. Lysyl Oxidase-Like 4 Fosters an Immunosuppressive Microenvironment During Hepatocarcinogenesis. Hepatology 2021;73:2326-2341.
9. Han J, Bai Y, Wang J, et al. REC8 promotes tumor migration, invasion and angiogenesis by targeting the PKA pathway in hepatocellular carcinoma. Clin Exp Med 2021;21:479-492.
10. Bendlová B, Vaňková M, Hill M, et al. ZBTB16 gene variability influences obesity-related parameters and serum lipid levels in Czech adults. Physiol Res 2017;66:S425-s31.
11. Palomer E, Martín-Segura A, Baliyan S, et al. Aging triggers a repressive chromatin state at bdnf promoters in hippocampal neurons. Cell Rep

2016;16:2889-2900.
12. Ahmed MY, Chioza BA, Rajab A, et al. Loss of PCLO function underlies pontocerebellar hypoplasia type III. Neurology 2015;84:1745-1750.
13. Kong F, Kong D, Yang X, et al. Integrative analysis of highly mutated genes in hepatitis B virus-related hepatic carcinoma. Cancer Med 2020;9:2462-2479.
14. Chiba T, Noji K, Shinozaki S, et al. Diet-induced non-alcoholic fatty liver disease affects expression of major cytochrome P450 genes in a mouse model. J Pharm Pharmacol 2016;68:1567-1576.
15. Sun Y, Chen ZY, Gan X, et al. A novel four-gene signature for predicting the prognosis of hepatocellular carcinoma. Scand J Gastroenterol 2022;57:1227-1237.
16. Schlingmann KP, Renigunta A, Hoorn EJ, et al. Defects in KCNJ16 cause a novel tubulopathy with hypokalemia, salt wasting, disturbed acid-base homeostasis, and sensorineural deafness. J Am Soc Nephrol 2021;32:1498-1512.
17. Xing S, Kan J, Su A, et al. The prognostic value of major facilitator superfamily domain-containing protein 2A in patients with hepatocellular carcinoma. Aging (Albany NY) 2019;11:8474-8483.
18. Katsyuba E, Mottis A, Zietak M, et al. De novo NAD^+ synthesis enhances mitochondrial function and improves health. Nature 2018;563:354-359.
19. Hassan HM, Isovic M, Kolendowski B, et al. Loss of thymine DNA glycosylase causes dysregulation of bile acid homeostasis and hepatocellular carcinoma. Cell Rep 2020;31:107475.

Table S3. Metabolites that classify diet, age, and FXR activity.

Metabolites (Specimens)	Source	Functions or Roles	Changes	Disease implications
1,5-anhydroglucitol (Liver)	Mainly from food, well absorbed in the intestine, and is distributed to all organs and tissues.	A glycemic marker	Western diet (Down)<br>Aging (Up)	Serum 1,5-anhydroglucitol concentrations are lower in cirrhotic and chronic liver disease patients than those in healthy people [1,2].<br>Low serum 1,5-anhydroglucitol concentration is a predictor of short-term mortality in Hepatitis B virus-related acute-on-chronic liver failure patients [3].
Linoleic acid (Liver)	Hydrogenated vegetable oils	An omega-6 trans fatty acid; α-linolenic acid and linoleic acid metabolism	Western diet (Down)	Dietary conjugated linoleic acid protects the liver from NAFLD in a rat model [4].
Squalene (Liver)	Human sebum (5%), fish liver oils, yeast lipids, and many vegetable oils (e.g., palm oil, cottonseed oil, rapeseed oil)	A biochemical precursor to the whole family of steroids; An intermediate for cholesterol synthesis; A precursor to phytosterol synthesis; A bactericide	Western diet (Up)<br>Aging (Up)<br>FXR KO (Down)	Squalene decreases hepatic cholesterol and triglycerides [5].
Heptadecanoic acid (Liver)	Exogenous origin, such as dairy fats (milk and meat)	A saturated fatty acid	Western diet (Up)	Biomarkers for dietary food (fat) intake assessment; Biomarkers for coronary heart disease risk and type II diabetes mellitus risk [6].

2-aminobutyric acid (Liver)	Biosynthesized by transamination of oxobutyrate; A metabolite in isoleucine biosynthesis; A non-essential amino acid that is primarily derived from the catabolism of methionine, threonine, and serine.	A non-proteinogenic α-amino acid	Western diet (Down)	-
Trimethylamine (Serum)	A product of decomposition of plants and animals; A bacteria metabolite	A colorless, hygroscopic, and flammable simple amine with a typical fishy odor in low concentrations; Ammonia like odor in higher concentrations.	Western diet (Down)	NAFLD patients have increased trimethylamine in the intestine and liver [7].
3-Hydroxyisobutyrate (Serum)	An intermediate in L-valine metabolism	Valine, leucine, and isoleucine degradation	Western diet (Down)<br>FXR KO (Down)	Plasma 3-hydroxyisobutyrate is a marker of hepatic mitochondrial fatty acid oxidation in male Wistar rats [8].
Aspartate (Serum)	Found in all organisms ranging from bacteria to plants to animals; Nonessential amino acid derived from glutamic acid by enzymes using vitamin B6.	Proteinogenic α-amino acid; Arginine and proline metabolism; Aspartate metabolism; Urea cycle	Western diet (Up)	Administration of L-aspartate in vitro or in mice efficiently ameliorates metabolic dysfunction-associated fatty liver disease [9].

Leucine (Serum)	Essential amino acid; Human dietary sources are foods that contain protein, such as meats, dairy products, soy products, beans and legumes.	A branched chain amino acid; Proteinogenic α-amino acid; Valine, leucine, and isoleucine degradation	Western diet (Up)	L-leucine supplementation is protective in patients with liver cirrhosis [10].
Histidine (Serum)	Essential amino acid; Exogenous food, such as fruits (berries)	α-amino acid; Histidine metabolism; Nitrogen metabolism; Beta-alanine metabolism; Anti-oxidant, anti-inflammatory and anti-secretory properties	Western diet (Up)	Histidine supplementation ameliorates metabolic syndrome [11].
Urea (Serum)	Exogenous food, such as berries	Urea cycle; Arginine and proline metabolism	Western diet (Down)<br>FXR KO (Down)	Urea cycle disorders [12].
2-Oxoisocaproate (Serum)	Endogenously produced metabolite	Valine, leucine, and isoleucine degradation; A neurotoxin and a metabotoxin.	Western diet (Up)	Plasma 2-oxoisocaproate (ketoleucine) concentrations are increased in patients with metabolic stress [13].
N-Methylhydantoin (Serum)	Exogenous, such as animal foods.	A bacterial metabolite; A imidazolidine-2,4-dione that is the N-methyl-derivative of hydantoin.	Western diet (Up)	-
Methionine (Serum)	Essential amino acid; Exogenous food such as	Proteinogenic α-amino acid; Glycine, serine, and	Western diet (Down)	NAFLD patients have low liver methionine concentrations [14].

	berries; An intermediate in transmethylation reactions	threonine metabolism; Methionine metabolism	FXR KO (Down)	
Asparagine (Serum)	Non-essential amino acid, the precursor to asparagine is oxaloacetate; Exogenous food such as berries	Proteinogenic α-amino acid; Aspartate metabolism; Nitrogen metabolism; Ammonia recycling	Western diet (Up)	Upregulating asparagine synthetase is beneficial for alleviating liver injury [15].
Sucrose (Urine)	From sugarcane (Saccharum officinarum), sugar beet (Beta vulgaris), and other plants	Sucrose is a sweetener and used in food products as a preservative, antioxidant, moisture control agent, stabilizer, and thickening agent; Galactose metabolism	Western diet (Up)	Excessive sucrose intake leads to NAFLD [16].
Trimethylamine (Urine)	Decomposition of plants and animals	A uremic toxin; A marker for urinary tract infection brought on by *E. coli*.	Western diet (Down)	Trimethylamine in the intestine and liver is increased in NAFLD patients [7].
Trimethylamine N-oxide (Urine)	Biosynthesized endogenously from trimethylamine, which is derived from choline.	A uremic toxin; TMAO alters cholesterol metabolism in the intestines, in the liver and in arterial wall.	Western diet (Down)	Trimethylamine-N-oxide promotes brain aging and cognitive impairment in mice [17].<br>TMAO supplementation aggravates liver steatosis [18].
Hippurate (Urine)	Produced from the metabolism of benzoate, which is mainly stored in the liver mitochondria.	Reflect hepatic function	Western diet (Up)	Elevated blood hippurate associates with improved hepatic steatosis and good metabolic health [19].

Pantothenate Vitamin B5 (Urine)	From everywhere and small quantities of pantothenic acid are found in nearly every food, with high amounts in whole grain cereals, legumes, eggs, meat, and royal jelly.	A water-soluble vitamin required to sustain life; Beta-alanine metabolism; Pantothenate and CoA biosynthesis	Western diet (Down)<br>FXR KO (Up)	-
Succinate semialdehyde (Liver)	An intermediate in the catabolism of gamma-aminobutyrate or gamma-aminobutyric acid	High levels of succinate semialdehyde function as a neurotoxin and a metabotoxin.	Aging (Up)	-
Xylitol (Liver)	Xylitol exists in all living species, ranging from bacteria to plants to humans.	Organism's growth, development, or reproduction	Aging (Up)	-
Valine (Liver)	Essential amino acid; Human dietary sources are foods that contain protein, such as meats, dairy products, soy products, beans and legumes.	Proteinogenic α-amino acid; Branched chain amino acid; Valine, leucine, and isoleucine degradation; Propanoate metabolism; Transcription/Translation	Aging (Up)	Excessive valine causes NAFLD [20].
Glyceric acid (Liver)	Obtained from oxidation of glycerol.	Glycine, serine, and threonine metabolism; Glycerolipid metabolism	Aging (Up)	Oral D-glyceric acid activates mitochondrial metabolism and reduces inflammation among 50-60-year-old healthy volunteers [21].

Aspartic acid (Liver)	Aspartic acid is found in all organisms ranging from bacteria to plants to animals.	Proteinogenic α-amino acid;<br>Transcription/translation;<br>Arginine and proline metabolism;<br>Aspartate metabolism	Aging (Down)	-
Ethanolamine (Liver)	Ethanolamine exists in all living species, ranging from bacteria to plants to humans.	An initial precursor for the biosynthesis of two primary phospholipid classes, phosphatidylcholine (PC) and phosphatidylethanolamine (PE).	Aging (Down)	Ethanolamine protects against hyperlipidemia in aged mice [22].
Glutaric acid (Liver)	Glutaric acid is naturally produced in the body during the metabolism of some amino acids, including lysine and tryptophan.	Glutaric acid may cause irritation to the skin and eyes. Glutaric acid acts as an acidogen and a metabotoxin when present in sufficiently high levels.	Aging (Up)	-
Ascorbic acid (Liver)	Ascorbic acid is found naturally in citrus fruits and many	Necessary to maintain connective tissue and bone.	Aging (Up)	Ascorbic acid inhibits obesity and nonalcoholic fatty liver disease [23].

	vegetables and is an essential nutrient in human diets.	Tyrosine metabolism		
2-aminobutyric acid (Liver)	A non-essential amino acid; Biosynthesized by transamination of oxobutyrate, a metabolite in isoleucine biosynthesis.	Non-proteogenic amino acid	Aging (Down)	-
2-monoolein (Liver)	A major end product of the intestinal digestion of dietary fats in animals via the enzyme pancreatic lipase.	Act as emulsifiers, helping to mix ingredients such as oil and water that would not otherwise blend well	Aging (Up)	-
3-(1-pyrazolyl)-l-alanine (Liver)	L-alanine derivative	A non-proteinogenic L-α-amino acid	Aging (Up)	-
Glycolic acid (Liver, serum)	A metabolite in bacteria such as Acetobacter, *Escherichia*	A nephrotoxin if consumed orally; A known inhibitor of tyrosinase; Renal toxicity	Aging (Up in the liver)<br>FXR KO (Down in serum)	-
Beta-glutamic acid (Liver)	A metabolite found in the aging mouse brain; A natural product found in Chondria armata	A marine metabolite and an algal metabolite.	Aging (Up)	-
Uridine (Liver)	Uridine can be synthesized from uracil. Uridine is found in many foods (anything containing RNA).	A nucleoside consisting of uracil and D-ribose and a component of RNA; Pyrimidine metabolism	Aging (Up)<br>FXR KO (Up)	Uridine alleviates carbon tetrachloride-induced liver fibrosis [24].

Serine (Liver)	Serine is found in all organisms ranging from bacteria to plants to animals.	Proteinogenic amino acid; Nitrogen metabolism; Starch and sucrose metabolism; Glycine, serine, and threonine metabolism	Aging (Down)	Reduced hepatic serine contributes to the development of fatty liver disease [25].
Palmitoleic acid (Liver)	A common constituent of the glycerides of human adipose tissue; From gut bacteria, such as Akkermansia muciniphila; Macadamia oil (Macadamia integrifolia) and sea buckthorn oil (Hippophae rhamnoides) are botanical sources of palmitoleic acid, containing 22 and 40% respectively.	A monounsaturated fatty acid; Reduce hepatic gluconeogenesis	Aging (Down)	Palmitoleic acid reduces high fat diet-induced liver inflammation [26]. Palmitoleic acid improves metabolic functions in fatty liver [27]. Palmitoleic acid protects against hypertension [28].
Lactic acid (Liver)	In animals, L-lactate is constantly produced from pyruvate via the enzyme lactate dehydrogenase in a process of fermentation during normal metabolism and exercise. Exogenous food such as beverages.	Gluconeogenesis; Pyruvate metabolism	Aging (Up)	-

Lithocholic acid (Liver)	It is formed from chenodeoxycholate by bacterial action and is usually conjugated with glycine or taurine.	Secondary bile acid; It acts as a detergent to solubilize fats for absorption.	Aging (Down)	Lithocholic acid inhibits inflammation and regulates metabolism [29].
Pyruvate (Serum)	Pyruvate is found in all living organisms ranging from bacteria to plants to humans. It is intermediate compound in the metabolism of carbohydrates, proteins, and fats.	Urea cycle; Glucose-alanine cycle; Glycine, serine, and threonine metabolism	Aging (Up)	-
1.3-Dihydroxyacetone (Serum)	It is often derived from plant sources such as sugar beets and sugar cane, by the fermentation of glycerin.	In combination with naphthoquinones, it acts as a sun screening agent.	Aging (Down)	-
Acetone (Serum)	Acetone is produced and disposed of in the human body through normal metabolic processes. It is normally present in blood and urine.	Ketone body metabolism	Aging (Down)<br>FXR KO (Down)	-
Methylamine (Urine)	Methylamine occurs endogenously from amine catabolism; Methylamines produced by microbial	Tyrosine metabolism	Aging (Down)	-

	metabolism of dietary choline and L-carnitine.<br>Exogenous food such as teas			
N.N-Dimethylglycine (Urine)	A derivative of the amino acid glycine	A microbial metabolite; A biomarker for the consumption of legumes	Aging (Down)<br>FXR KO (Up)	-
Betaine (Urine)	Exogenous food such as fruits (e.g., berries)	Glycine, serine, and threonine metabolism; Methionine metabolism	Aging (Up)	Betaine inhibits inflammation and apoptosis [30].
2-Hydroxyisobutyrate (Urine)	Non-essential, secondary metabolite	May serve as defense or signaling molecules.	Aging (Up)<br>FXR KO (Up)	-
Sn-Glycero-3-phosphocholine (Urine)	Formed in the breakdown of phosphatidylcholine	One of the four major organic osmolytes in renal medullary cells, changing their intracellular osmolyte concentration in parallel with extracellular tonicity during cellular osmoadaptation.<br>Retinol metabolism	Aging (Up)<br>FXR KO (Up)	-
3-Indoxylsulfate (Urine)	A metabolite of the common amino acid tryptophan and is derived through the consumption, digestion, and	A uremic toxin and cardiotoxin	Aging (Up)	-

	microbial processing of protein-rich foods. Indoxyl sulfate is technically a bacterial co-metabolite, meaning that it is derived from both bacterial and host metabolism.			
Ascorbate<br>Vitamin C<br>(Urine)	An essential nutrient in human diets, including citrus fruits and many vegetables;<br>A microbial metabolite produced by Ketogulonicigenium	A water-soluble vitamin; Antioxidant; An electron donor for enzymes involved in collagen hydroxylation, biosynthesis of carnitine and norepinephrine.	Aging (Down)	Ascorbate ameliorates factors linked to Alzheimer's disease pathogenesis [31].
Melibiose<br>(Liver)	This sugar is produced and metabolized only by enteric and lactic acid bacteria and other microbes, such as *Escherichia*. It is not an endogenous metabolite but may be obtained from the consumption of partially fermented molasses, brown sugar, or honey.	Galactose metabolism	Aging (Up),<br>FXR KO (Down)	-
Glucoheptulose<br>(Liver)	L-arabinose	-	FXR KO (Down)	-

UDP-N-acetylglucosamine (Liver)	Exogenous, food such as animal food	Glucose sensor; Amino sugar metabolism; Elevated UDP-N-acetylglucosamine has an effect on insulin-stimulated glucose uptake.	FXR KO (Up)	-
Uridine (Liver)	Synthesized from uracil; Uridine is found in many foods (anything containing RNA).	Pyrimidine metabolism	FXR KO (Up)	Uridine alleviates carbon tetrachloride induced liver fibrosis [24].
Isomaltose (Liver)	A product of the caramelization of glucose; Exogenous, food such as beverages	Starch and sucrose metabolism; Metabolic pathways	FXR KO (Up)	Isomaltulose improves insulin resistance in NAFLD patients [32].
Ribose-5-phosphate (Liver)	Exogenous, food such as fruits; A product and an intermediate of the pentose phosphate pathway.	Purine metabolism; Pentose phosphate pathway	FXR KO (Down)	Ribose-5-phosphate isomerase A overexpression induces oncogenesis in HCC [33].
2-hydroxybutanoic acid (Liver)	Primarily produced in mammalian hepatic tissues that catabolize L-threonine or synthesize glutathione. Exogenous, food such as animal foods	An early marker for both insulin resistance and impaired glucose regulation	FXR KO (Down)	-

Arachidic acid (Liver)	A minor constituent of butter, perilla oil, peanut oil, corn oil, and cocoa butter.	Saturated, long-chain fatty acids	FXR KO (Down)	Arachidic acid induces liver dysfunction in hyperglycaemic rats [34].
Malic acid (Liver, serum)	Exogenous, food such as herbs and spices	Citric acid cycle; Gluconeogenesis; Pyruvate metabolism; Malate-aspartate shuttle	FXR KO (Up in the liver) FXR KO (Down in the serum)	Malic acid is increased in NAFLD [35]
Succinate (Serum, Urine)	Exogenous, food such as fruits	A cell signaling molecule; Succinate alters gene expression patterns, thereby modulating the epigenetic landscape or it can exhibit hormone-like signaling functions.	FXR KO (Down in serum and up in urine)	Elevated extracellular succinate in liver tissue drives inflammation [36].
Alanine (Serum)	It is formed in vivo by the degradation of dihydrouracil and carnosine.	A neurotoxin; A mitochondrial toxin; A metabotoxin; Beta-alanine metabolism	FXR KO (Down)	-
Glutamine (Serum)	Non-essential amino acid	Proteinogenic α-amino acid; Glutamate metabolism; Purine metabolism; Urea cycle	FXR KO (Up)	Glutamine is beneficial for human inflammatory bowel disease [37].
Phenylalanine (Serum)	An essential amino acid and the precursor of the amino acid tyrosine, highly concentrated in	Proteinogenic α-amino acid; A precursor for catecholamines including	FXR KO (Up)	-

	high protein foods, such as meat, cottage cheese, and wheat germ. An additional dietary source of phenylalanine is artificial sweeteners containing aspartame.	tyramine, dopamine, epinephrine, and norepinephrine; A neurotoxin and a metabotoxin.		
Tyrosine (Serum)	Exogenous, food such as fruits	Proteinogenic α-amino acid; Tyrosine metabolism	FXR KO (Down)	-
Glucose (Serum)	Exogenous, food such as fruits	Primary source of energy for all living organisms, Glycolysis; Gluconeogenesis; Lactose synthesis	FXR KO (Up)	-
Creatinine (Urine)	Exogenous, food such as herbs and spices	An amino acid derivative; A waste product and is normally eliminated in large quantities by the kidneys through urinary excretion.	FXR KO (Down)	-
Taurine (Urine)	An essential amino acid; Foods such as vegetables, animal and fish protein	A neurotransmitter in the brain; Taurine and hypotaurine metabolism; Primary bile acid biosynthesis	FXR KO (Down)	Taurine displays potential ameliorating effects against different neurological disorders [38].

N-Phenylacetylglycine (Urine)	Exogenous, food such as animal food	A surrogate biomarker for phospholipidosis	FXR KO (Up)	A biomarker for dimethylnitrosamine-induced hepatic fibrosis in a rat model [39].
Guanidoacetate (Urine)	Exists naturally in all vertebrates; Formed primarily in the kidneys by transferring the guanidine.	α-amino acid and derivatives; Arginine and proline metabolism; Glycine, serine, and threonine metabolism	FXR KO (Down)	-
trans-Aconitate (Urine)	Normally present in human urine; Detected in foods, such as garden tomato fruits, root vegetables, soybeans, and rice.	A biomarker for the consumption of soy products	FXR KO (Down)	-
Cis-Aconitate (Urine)	Cow milk	Glutaminolysis and cancer pathway; Citric acid cycle	FXR KO (Down)	-
N.N-Dimethylglycine (Urine)	Exogenous, food such as animal foods; An amino acid derivative found in the cells of all plants and animals and can be obtained in the diet in small amounts from grains and meat; A byproduct of homocysteine metabolism; A microbial metabolite	A biomarker for the consumption of legumes; Glycine, serine, and threonine metabolism; Methionine metabolism	FXR KO (Up)	Plasma N.N-Dimethylglycine is markedly decreased in Alzheimer's disease patients compared with normal controls [40].

HCC, hepatocellular carcinoma; NAFLD, nonalcoholic liver disease; -, unknown.

References

1. Yamagishi S, Ohta M. Serum 1,5-anhydro-D-glucitol levels in liver cirrhosis. Acta Diabetol 1998;35:65-66.
2. Koga M, Murai J, Saito H, et al. 1,5-Anhydroglucitol levels are low irrespective of plasma glucose levels in patients with chronic liver disease. Ann Clin Biochem 2011;48:121-125.
3. Zhang L, Zhao Y, Xie Z, et al. 1,5-Anhydroglucitol Predicts Mortality in Patients with HBV-Related Acute-on-chronic Liver Failure. J Clin Transl Hepatol 2022;10:651-659.
4. Nagao K, Inoue N, Wang Y-M, et al. Dietary conjugated linoleic acid alleviates nonalcoholic fatty liver disease in Zucker (fa/fa) rats. The Journal of nutrition 2005;135:9-13.
5. Lou-Bonafonte JM, Martínez-Beamonte R, Sanclemente T, et al. Current insights into the biological action of squalene. Mol Nutr Food Res 2018:e1800136.
6. Jenkins B, West JA, Koulman A. A review of odd-chain fatty acid metabolism and the role of pentadecanoic Acid (c15:0) and heptadecanoic Acid (c17:0) in health and disease. Molecules 2015;20:2425-2444.
7. Albillos A, de Gottardi A, Rescigno M. The gut-liver axis in liver disease: Pathophysiological basis for therapy. J Hepatol 2020;72:558-577.
8. Bjune MS, Lindquist C, Hallvardsdotter Stafsnes M, et al. Plasma 3-hydroxyisobutyrate (3-HIB) and methylmalonic acid (MMA) are markers of hepatic mitochondrial fatty acid oxidation in male Wistar rats. Biochim Biophys Acta Mol Cell Biol Lipids 2021;1866:158887.
9. Rao Y, Kuang Z, Li C, et al. Gut Akkermansia muciniphila ameliorates metabolic dysfunction-associated fatty liver disease by regulating the metabolism of L-aspartate via gut-liver axis. Gut Microbes 2021;13:1-19.
10. Dos Santos ALS, Anastácio LR. The impact of L-branched-chain amino acids and L-leucine on malnutrition, sarcopenia, and other outcomes in patients with chronic liver disease. Expert Rev Gastroenterol Hepatol 2021;15:181-194.
11. DiNicolantonio JJ, McCarty MF, JH OK. Role of dietary histidine in the prevention of obesity and metabolic syndrome. Open Heart 2018;5:e000676.
12. Nagamani SCS, Ali S, Izem R, et al. Biomarkers for liver disease in urea cycle disorders. Mol Genet Metab 2021;133:148-156.
13. Trupp M, Jonsson P, Ohrfelt A, et al. Metabolite and peptide levels in plasma and CSF differentiating healthy controls from patients with newly diagnosed Parkinson's disease. J Parkinsons Dis 2014;4:549-560.
14. Wang H, Wu Y, Tang W. Methionine cycle in nonalcoholic fatty liver disease and its potential applications. Biochem Pharmacol 2022;200:115033.

15. Wang S, Ding Y, Dong R, et al. Canagliflozin improves liver function in rats by upregulating asparagine synthetase. Pharmacology 2021;106:606-615.
16. Chung M, Ma J, Patel K, et al. Fructose, high-fructose corn syrup, sucrose, and nonalcoholic fatty liver disease or indexes of liver health: a systematic review and meta-analysis. Am J Clin Nutr 2014;100:833-849.
17. Li D, Ke Y, Zhan R, et al. Trimethylamine-N-oxide promotes brain aging and cognitive impairment in mice. Aging Cell 2018;17:e12768.
18. Tan X, Liu Y, Long J, et al. Trimethylamine N-Oxide aggravates liver steatosis through modulation of bile acid metabolism and inhibition of farnesoid x receptor signaling in nonalcoholic fatty liver disease. Mol Nutr Food Res 2019;63:e1900257.
19. Lee KS, Cho Y, Kim H, et al. Association of metabolomic change and treatment response in patients with non-alcoholic fatty liver disease. Biomedicines 2022;10.
20. Jian H, Miao S, Liu Y, et al. Dietary valine ameliorated gut health and accelerated the development of nonalcoholic fatty liver disease of laying hens. Oxid Med Cell Longev 2021;2021:4704771.
21. Hirvonen OP, Lehti M, Kyröläinen H, et al. Heme oxygenase-1 and blood bilirubin are gradually activated by oral D-glyceric acid. Antioxidants (Basel) 2022;11.
22. Ding L, Zhang L, Shi H, et al. The protective effect of dietary epa‐enriched ethanolamine plasmalogens against hyperlipidemia in aged mice. European Journal of Lipid Science and Technology 2020;122:2000179.
23. Lee H, Ahn J, Shin SS, et al. Ascorbic acid inhibits visceral obesity and nonalcoholic fatty liver disease by activating peroxisome proliferator-activated receptor α in high-fat-diet-fed C57BL/6J mice. Int J Obes (Lond) 2019;43:1620-30.
24. Zheng WV, Li Y, Cheng X, et al. Uridine alleviates carbon tetrachloride-induced liver fibrosis by regulating the activity of liver-related cells. J Cell Mol Med 2022;26:840-854.
25. Sim WC, Lee W, Sim H, et al. Downregulation of PHGDH expression and hepatic serine level contribute to the development of fatty liver disease. Metabolism 2020;102:154000.
26. Souza CO, Teixeira AAS, Biondo LA, et al. Palmitoleic acid reduces high fat diet-induced liver inflammation by promoting PPAR-γ-independent M2a polarization of myeloid cells. Biochim Biophys Acta Mol Cell Biol Lipids 2020;1865:158776.
27. de Souza CO, Teixeira AAS, Biondo LA, et al. Palmitoleic acid improves metabolic functions in fatty liver by PPARα-dependent AMPK activation. J Cell Physiol 2017;232:2168-2177.
28. Tang J, Yang B, Yan Y, et al. Palmitoleic acid protects against hypertension by inhibiting NF-κB-mediated inflammation. Mol Nutr Food Res

2021;65:e2001025.
29. Shao J, Ge T, Tang C, et al. Synergistic anti-inflammatory effect of gut microbiota and lithocholic acid on liver fibrosis. Inflamm Res 2022;71:1389-1401.
30. Veskovic M, Mladenovic D, Milenkovic M, et al. Betaine modulates oxidative stress, inflammation, apoptosis, autophagy, and Akt/mTOR signaling in methionine-choline deficiency-induced fatty liver disease. Eur J Pharmacol 2019;848:39-48.
31. Monacelli F, Acquarone E, Giannotti C, et al. Vitamin C, Aging and Alzheimer's Disease. Nutrients 2017;9.
32. Kawaguchi T, Nakano D, Oriishi T, et al. Effects of isomaltulose on insulin resistance and metabolites in patients with non-alcoholic fatty liver disease: A metabolomic analysis. Mol Med Rep 2018;18:2033-2042.
33. Ciou SC, Chou YT, Liu YL, et al. Ribose-5-phosphate isomerase A regulates hepatocarcinogenesis via PP2A and ERK signaling. Int J Cancer 2015;137:104-115.
34. Moisés Laparra J, Díez-Municio M, Javier Moreno F, et al. Kojibiose ameliorates arachidic acid-induced metabolic alterations in hyperglycaemic rats. Br J Nutr 2015;114:1395-1402.
35. Zhu MN, Zhao CZ, Wang CZ, et al. Dataset for liver metabolomic profile of highland barley Monascus purpureus went extract-treated golden hamsters with nonalcoholic fatty liver disease. Data Brief 2022;40:107773.
36. Mills EL, Harmon C, Jedrychowski MP, et al. UCP1 governs liver extracellular succinate and inflammatory pathogenesis. Nat Metab 2021;3:604-617.
37. Kim MH, Kim H. The Roles of Glutamine in the Intestine and its implication in intestinal diseases. Int J Mol Sci 2017;18.
38. Jakaria M, Azam S, Haque ME, et al. Taurine and its analogs in neurological disorders: Focus on therapeutic potential and molecular mechanisms. Redox Biol 2019;24:101223.
39. Ju HK, Chung HW, Lee HS, et al. Investigation of metabolite alteration in dimethylnitrosamine-induced liver fibrosis by GC-MS. Bioanalysis 2013;5:41-51.
40. Wang G, Zhou Y, Huang FJ, et al. Plasma metabolite profiles of Alzheimer's disease and mild cognitive impairment. J Proteome Res 2014;13:2649-2658.
